# Multisystem involvement, defective lysosomes and impaired autophagy in a novel rat model of nephropathic cystinosis

**DOI:** 10.1093/hmg/ddac033

**Published:** 2022-02-08

**Authors:** Patrick Krohn, Laura Rita Rega, Marianne Harvent, Beatrice Paola Festa, Anna Taranta, Alessandro Luciani, Joseph Dewulf, Alessio Cremonesi, Francesca Diomedi Camassei, James V M Hanson, Christina Gerth-Kahlert, Francesco Emma, Marine Berquez, Olivier Devuyst

**Affiliations:** Institute of Physiology, University of Zurich, Zurich 8057, Switzerland; Renal Diseases Research Unit, Genetics and Rare Diseases Research Area, Bambino Gesù Children’s Hospital, IRCCS, Rome 00165, Italy; Institute of Physiology, University of Zurich, Zurich 8057, Switzerland; Institute of Physiology, University of Zurich, Zurich 8057, Switzerland; Renal Diseases Research Unit, Genetics and Rare Diseases Research Area, Bambino Gesù Children’s Hospital, IRCCS, Rome 00165, Italy; Institute of Physiology, University of Zurich, Zurich 8057, Switzerland; Department of Laboratory Medicine, Cliniques universitaires Saint Luc, UCLouvain, Brussels 1200, Belgium; Department of Biochemistry, de Duve Institute, UCLouvain, Brussels 1200, Belgium; Division of Clinical Chemistry and Biochemistry, University Children’s Hospital Zurich, Zurich 8032, Switzerland; Department of Laboratories–Pathology Unit, Bambino Gesù Children’s Hospital, Rome 00165, Italy; Department of Ophthalmology, University Hospital Zurich and University of Zurich, Zurich 8091, Switzerland; Department of Ophthalmology, University Hospital Zurich and University of Zurich, Zurich 8091, Switzerland; Renal Diseases Research Unit, Genetics and Rare Diseases Research Area, Bambino Gesù Children’s Hospital, IRCCS, Rome 00165, Italy; Department of Pediatric Subspecialties, Division of Nephrology, Children’s Hospital Bambino Gesù, IRCCS, Rome 00165, Italy; Institute of Physiology, University of Zurich, Zurich 8057, Switzerland; Institute of Physiology, University of Zurich, Zurich 8057, Switzerland; Institut de Recherche Expérimentale et Clinique, UCLouvain, Brussels, Belgium

## Abstract

Recessive mutations in the *CTNS* gene encoding the lysosomal transporter cystinosin cause cystinosis, a lysosomal storage disease leading to kidney failure and multisystem manifestations. A *Ctns* knockout mouse model recapitulates features of cystinosis, but the delayed onset of kidney manifestations, phenotype variability and strain effects limit its use for mechanistic and drug development studies. To provide a better model for cystinosis, we generated a *Ctns* knockout rat model using CRISPR/Cas9 technology. The *Ctns*^−/−^ rats display progressive cystine accumulation and crystal formation in multiple tissues including kidney, liver and thyroid. They show an early onset and progressive loss of urinary solutes, indicating generalized proximal tubule dysfunction, with development of typical swan-neck lesions, tubulointerstitial fibrosis and kidney failure, and decreased survival. The *Ctns*^−/−^ rats also present crystals in the cornea, and bone and liver defects, as observed in patients. Mechanistically, the loss of cystinosin induces a phenotype switch associating abnormal proliferation and dedifferentiation, loss of apical receptors and transporters, and defective lysosomal activity and autophagy in the cells. Primary cultures of proximal tubule cells derived from the *Ctns*^−/−^ rat kidneys confirmed the key changes caused by cystine overload, including reduced endocytic uptake, increased proliferation and defective lysosomal dynamics and autophagy. The novel *Ctns*^−/−^ rat model and derived proximal tubule cell system provide invaluable tools to investigate the pathogenesis of cystinosis and to accelerate drug discovery.

## Introduction

The proximal tubule (PT) segment of the kidney reabsorbs and processes large quantities of essential nutrients and solutes, playing a crucial role in maintaining homeostasis. These transport processes are sustained by an active endolysosomal system operating at the apical pole of the cells. Congenital or acquired disorders affecting the endolysosomes lead to PT dysfunction, characterized by the inappropriate loss of low molecular weight (LMW) proteins and solutes in the urine. This condition, referred to as renal Fanconi syndrome (RFS), may lead to severe electrolyte imbalance, growth and bone defects, and the development of chronic kidney disease (CKD) (1).

The leading cause of inherited RFS in children is nephropathic cystinosis (MIM #219800), a lysosomal storage disease (LSD) caused by recessive, inactivating mutations in the *CTNS* gene coding for the proton-driven transporter cystinosin that exports cystine out of lysosomes (2). The loss of cystinosin results in the accumulation of cystine within lysosomes in all organs, but particularly affecting the PT segment of the kidney. Infants with cystinosis show manifestations of PT dysfunction and RFS within the first year of life, complicated by growth retardation, metabolic bone disease and progressing to CKD and kidney failure by the age of 10 years if untreated. Other complications resulting from cystine crystals and lysosomal disease include reduced vision and recurrent corneal erosions, hypothyroidism, hypogonadism, diabetes, myopathy, liver disease and degeneration of the central nervous system (3,4). The oral administration of cysteamine, which depletes cystine out from the lysosomes, delays the progression of kidney failure and improves the overall prognosis. However, cysteamine is poorly tolerated and does not treat or prevent PT dysfunction (3,5–7). Thus, there is an urgent need to identify novel treatment modalities for cystinosis.

Animal models are crucial to understand mechanisms of disease and to develop new therapies. The most used model for cystinosis is the *Ctns* knockout (*Ctns*^−/−^) mouse developed by Cherqui *et al.* (8), which recapitulates cystine overload, PT dysfunction and ocular alterations (9,10). Studies based on *Ctns*^−/−^ mice have identified the role of impaired endolysosomal trafficking and proteolysis, defective lysosomal clearance and apical dedifferentiation in disease progression (9,11–13). However, the *Ctns*^−/−^ mice present a less severe phenotype compared with cystinotic patients, with a late onset and high variability in the extent of tubular dysfunction, no kidney failure and no change in lifespan (7,10,14). Moreover, the kidney phenotype depends on the genetic background, with C57BL/6 *Ctns*^−/−^ mice showing cystine accumulation associated with kidney lesions, while FVB/N *Ctns*^−/−^ mice do not develop kidney disease (10,15). An effect of sex on cystine content (higher levels in female versus male kidneys) has also been observed, while no evidence for such an effect has been detected in humans (16). Discrepancies in diverse forms of autophagy between human- and mouse-derived samples have also been reported (11,17,18). More generally, mouse models have failed in many cases to be predictive due to evolutionary differences with humans (19).

As a model organism, the rat offers a high level of genomic and physiologic similarities with humans (20,21). Many inbred rat strains have been used in comparative physiology, cardiovascular, neurophysiology and behavioral studies, as well as for toxicology testing (22). One of these strains, the Long-Evans Agouti (LEA/Tohm) rat, used as a model of type 2 diabetes, was recently shown to carry a 13-bp deletion in the *Ctns* gene, causing accumulation of cystine in various tissues and the appearance of glycosuria and kidney tubular lesions before the onset of diabetes (23). The advantages of rats over mouse models include larger body size, allowing more detailed phenotyping and extensive sampling, and metabolic and detoxification pathways that are closer to humans (20,24–26). Since the first generation of knockout rats using embryo microinjection of zinc-finger nucleases (27) or homologous recombination in ES cells (28,29), genetically engineered rat models have provided competitive advantages for modeling human genetic diseases (30).

Here, we present a novel *Ctns* knockout rat model for nephropathic cystinosis obtained by using CRISPR/Cas9 (clustered regularly interspaced short palindromic repeats/CRISPR associated protein 9) technology. The *Ctns* knockout rats recapitulate essential clinical and molecular features of cystinosis, including the role of defective endolysosomal dynamics and autophagy. These features were verified in a primary cell culture system derived from the rat kidney. This rat model represents a powerful new tool to substantiate and to accelerate drug discovery in cystinosis.

## Results

### CRISPR/Cas9-induced deletion of *Ctns* and cystine accumulation in *Ctns*^−/−^ rats

**Figure 1:**
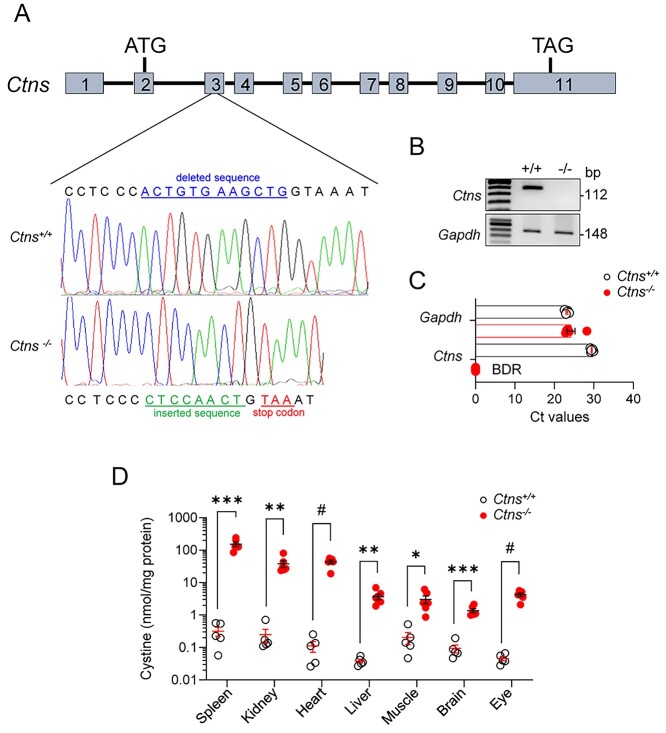
Generation and validation of the *Ctns* knockout rat model. (**A**) CRISPR/Cas9 induced a 12 bp deletion (blue), which was repaired by an 8 bp insertion (green), generating a frameshift of the open reading frame and resulting in a premature stop codon (TGA, red) in exon 3 of the *Ctns* gene. (**B**) *Ctns* genomic DNA analyzed by PCR and agarose gel electrophoresis isolated from kidney biopsies. (**C**) *Ctns* and *Gapdh* expression in kidney biopsies were analyzed by RT-qPCR (*n* = 5 rats per condition). (**D**) Cystine levels measured by HPLC in different tissues of 40-week-old *Ctns* rats (*n* = 5 *Ctns*^+/+^ and *n* = 6 *Ctns*^−/−^ rats per condition). Plotted data represent mean ± SEM. Each dot represents one rat. Two-tailed unpaired Student’s *t*-test, ^*^P < 0.05, ^*^^*^P < 0.01, ^*^^*^^*^P < 0.001 and #*P* < 0.0001 relative to *Ctns*^+/+^ rats. BDR: below detection range.

A rat model for cystinosis was generated by targeted disruption of the *Ctns* gene using CRISPR/Cas9. Injection of the two guide RNAs in oocytes of Sprague–Dawley rats caused a premature stop codon in exon 3 of *Ctns* (Fig. 1A). The deletion of *Ctns*/cystinosin was confirmed at DNA (Fig. 1B) and mRNA (Fig. 1C) levels. Screening for off-target sequences using CAS-OFFinder showed no editing in other genes (31). All rats were born at Mendelian ratio and were viable. The direct consequence of cystinosin deletion was reflected by elevated cystine content in multiple tissues including spleen, kidney, heart, liver, muscle, brain and eyes (Fig. 1D). These data validate the specific deletion of the *Ctns* gene by CRISPR/Cas9 and confirm the resulting, systemic accumulation of cystine in this novel rat model.

### *Ctns* ^−/−^ rats show growth retardation and proximal tubule dysfunction

**Figure 2:**
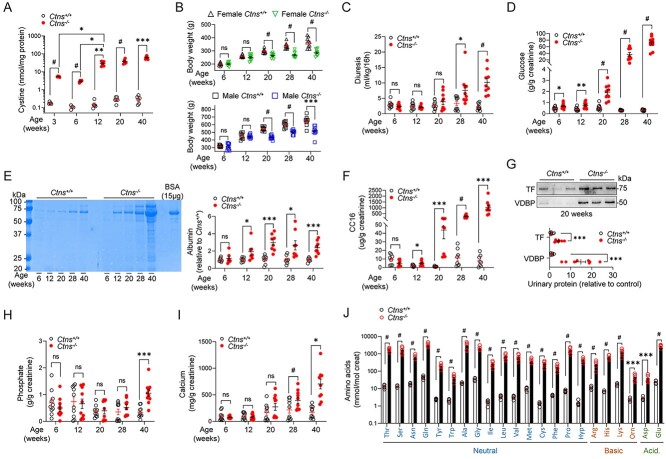
Deletion of *Ctns* leads to cystine accumulation in kidneys, growth retardation and renal Fanconi syndrome in rats. (**A**) Cystine levels measured by HPLC in kidney cortex from *Ctns*^+/+^ and *Ctns*^−/−^ rats at 3, 6, 12, 20 and 40 weeks of age (*n* = 5 rats at 3, *n* = 4 rats at 6 and *n* = 6 rats at 12, 20 and 40 weeks per group). (**B**) Measurement of body weight over time in male and female rats (*n* = 10 rats per group). (**C**) Overnight urine excretion (milliliters of urine per 16 h normalized to body weight; *n* = 10 rats per group). (**D**) Urinary excretion of glucose (*n* = 10 rats per group). (**E**) Coomassie blue–stained SDS-PAGE analysis of urine at 6, 12, 20, 28 and 40 weeks of age and densitometry quantification of albumin (*n* = 8 rats per group). A total of 15 μg of BSA was loaded as a positive control (Mw ∼ 66.5 kDa). (**F**) Urinary excretion of the low-molecular-weight protein CC16 (*n* = 8 rats per condition). (**G**) Representative western blotting and densitometry quantification of transferrin (TF) and vitamin D–binding protein (VDBP) in urine derived from 20-week-old *Ctns* rats (*n* = 8 rats per group). Measurement of urinary (**H**) phosphate and (**I**) calcium (*n* = 10 rats per group). (**J**) Relative concentration of amino acids in urine derived from 28-week-old *Ctns* rats (*n* = 5 rats per group). Ala, alanine; Arg, arginine; Asn, asparagine; Asp, aspartate; Cys, cysteine; Gln, glutamine; Glu, glutamate; Gly, glycine; His, histidine; Hyp, hydroxyproline; Ile, isoleucine; Leu, leucine; Lys, lysine; Met, methionine; Orn, ornithine; Phe, phenylalanine; Pro, proline; Ser, serine; Thr, threonine; Trp, tryptophan; Tyr, tyrosine; Val, valine. All the urine parameters were normalized to urinary creatinine concentration. Plotted data represent mean ± SEM. Each dot represents one rat. Two-tailed unpaired Student’s *t*-test, ^*^P < 0.05, ^*^^*^P < 0.01, ^*^^*^^*^P < 0.001 and *#P <* 0.0001 relative to *Ctns*^+/+^ or *Ctns*^−/−^ rats. ns: not significant.

We first characterized the kidney phenotype of the *Ctns* rats over time. *Ctns*^−/−^ animals displayed a progressive cystine accumulation in the kidneys from 3 weeks of age, compared with *Ctns*^+/+^ rats (Fig. 2A). Growth retardation was observed starting from 20 weeks in both males and females (Fig. 2B). Metabolic cage analyses revealed polyuria at 28 weeks in *Ctns*^−/−^ rats, paralleled by an increase in water consumption (Fig. 2C; Table 1). Blood and urine electrolyte levels were measured at different time points (Table 1; Fig. 2; Supplementary Material, Fig. S1 and Table S1).

**Table 1:** Body weight, urine and blood parameters in *Ctns* rats

	12 weeks male		12 weeks female		20 weeks male		20 weeks female		40 weeks male		40 weeks female	
	*Ctns* ^+/+^	*Ctns* ^−/−^	*Ctns* ^+/+^	*Ctns* ^−/−^	*Ctns* ^+/+^	*Ctns* ^−/−^	*Ctns* ^+/+^	*Ctns* ^−/−^	*Ctns* ^+/+^	*Ctns* ^−/−^	*Ctns* ^+/+^	*Ctns* ^−/−^
Body weight (g) *n* = 10	449 ± 12.3	447 ± 7.06	260 ± 3.10	249 ± 7.17	547 ± 8.41	434 ± 25.8^#^	298 ± 4.67	263 ± 5.02^#^	645 ± 19.0	517 ± 62.4^^*^^*^^*^^	363 ± 11.67	286 ± 7.22^#^
Water intake (ml/16 h) *n* = 10	43.4 ± 6.68	39.4 ± 2.71	31.7 ± 2.97	27.7 ± 1.16	37.2 ± 4.26	30.8 ± 1.72	42.4 ± 6.48	43.3 ± 2.89	31.1 ± 2.16	88.1 ± 15.6^^*^^*^^	40.0 ± 2.39	82.1 ± 6.36^^*^^*^^*^^
Diuresis (ml kg^−1^ BW/16 h) *n* = 5	2.11 ± 0.61	1.91 ± 0.17	2.71 ± 0.33	2.25 ± 0.33	1.96 ± 0.49	1.49 ± 0.20	3.51 ± 0.50	6.28 ± 0.84^*^	1.48 ± 0.22	8.39 ± 1.62 ^^*^^*^^	2.88 ± 0.43	12.4 ± 1.74^^*^^*^^*^^
U CC16 (μg g^−1^ creatinine) *n* = 4	2.2 ± 0.62	6.54 ± 1.8	1.69 ± 0.6	3,3 ± 0,39	1.3 ± 0.28	64.4 ± 10.3^^*^^*^^*^^	0.83 ± 0.1	13.5 ± 1.1^#^	3.82 ± 1.57	762 ± 237^*^	11.6 ± 3.7	1286 ± 334^^*^^*^^
U Glucose (g g^−1^ creatinine) *n* = 5	0.49 ± 0.08	0.94 ± 0.11^^*^^	0.32 ± 0.03	0.55 ± 0.07^^*^^	0.67 ± 0.13	2.28 ± 0.28^^*^^*^^*^^	0.42 ± 0.05	1.50 ± 0.34^^*^^*^^	0.35 ± 0.03	63.1 ± 9.45^^*^^*^^*^^	0.29 ± 0.02	79.6 ± 6.49^#^
U Phosphorus (g g^−1^ creatinine) *n* = 5	0.23 ± 0.10	0.16 ± 0.05	0.71 ± 0.12	0.78 ± 0.03	0.24 ± 0.05	0.06 ± 0.03	0.49 ± 0.09	0.72 ± 0.06	0.18 ± 0.04	0.82 ± 0.13^^*^^*^^	0.50 ± 0.07	1.28 ± 0.18^^*^^*^^
U Calcium (mg g^−1^ creatinine) *n* = 5	43.5 ± 8.9	38.9 ± 7.8	151 ± 12.1	122 ± 18.7	32.6 ± 9.4	80.8 ± 5.2^^*^^*^^	291.3 ± 48.9	465 ± 42.3^^*^^	67.7 ± 10.4	648 ± 101^^*^^*^^*^^	270 ± 24.1	758 ± 165^^*^^
U Creatinine (mg dl^−1^) *n* = 5	98.8 ± 20.2	93.7 ± 9.52	75.3 ± 10.0	75.4 ± 8.26	115 ± 25.0	119 ± 13.5	58.6 ± 10.8	33.3 ± 4.21	113 ± 17.6	27.6 ± 3.72^^*^^*^^	69.2 ± 9.80	27.4 ± 6.24^^*^^*^^
BUN (mg dl^−1^) *n* = 5	13.2 ± 0.45	12.8 ± 0.95	14.49 ± 1.00	13.2 ± 0.93	16.5 ± 0.67	16.73 ± 0.77	16.5 ± 0.94	16.7 ± 0.75	17.7 ± 1.67	19.8 ± 2.20	16.9 ± 1.12	16.1 ± 1.12
Enzymatic creatinine (mg dl^−1^) *n* = 5	0.24 ± 0.01	0.23 ± 0.04	0.33 ± 0.02	0.34 ± 0.02	0.27 ± 0.02	0.31 ± 0.06	0.30 ± 0.04	0.40 ± 0.04	0.32 ± 0.02	0.55 ± 0.03^^*^^*^^*^^	0.37 ± 0.01	0.50 ± 0.05^^*^^

BW, Body weight U, Urine; BUN, Blood Urea Nitrogen. All the measurements were performed on *Ctns*^+/+^ and *Ctns*^−/−^ male and female rats matched per age. Two-tailed unpaired Student’s *t*-test was applied between genotypes at the indicated time points. ^*^P < 0.05, ^*^^*^P < 0.01, ^*^^*^^*^P < 0.001 and *^#^P <* 0.0001 relative to *Ctns*^+/+^ rats.

Manifestations of PT dysfunction appeared early in *Ctns*^−/−^ rats with glycosuria starting at 6 weeks of age (Fig. 2D), followed by urinary loss of albumin and LMW proteins Clara cell secretory protein 16 (CC16), transferrin (TF) and vitamin D–binding protein (VDBP), starting at 12 weeks (Fig. 2E–G, Table 1). The LMW proteinuria increased over time (Table 1). Other tubular manifestations included an excessive loss of calcium and phosphate appearing at 28 and 40 weeks, respectively (Fig. 2H and I), in line with the hypocalcemia, hypophosphatemia and the loss of VDBP observed in *Ctns*^−/−^ rats (Fig. 2G; Supplementary Material, Fig. S1A–D; Table S1). A selective aminoaciduria, including glutamine, methionine and hydroxyproline was detected at 20 weeks in *Ctns*^−/−^ rats (Supplementary Material, Fig. S1E), evolving into a massive, generalized aminoaciduria at 28 weeks (Fig. 2J). No sex differences in PT dysfunction were observed in *Ctns*^−/−^ animals. The growth retardation, early PT dysfunction and development of renal Fanconi syndrome in *Ctns*^−/−^ rats faithfully reflect the situation observed in patients with nephropathic cystinosis.

### *Ctns* ^−/−^ rats develop tubular damage, kidney fibrosis and swan-neck lesions

**Figure 3:**
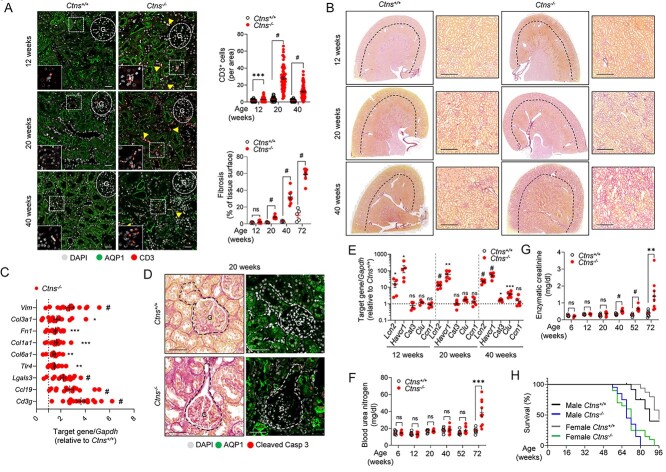
Inflammation, fibrosis and apoptosis in *Ctns*^−/−^ rat kidneys. (**A**) Representative confocal micrographs and quantification of the number of CD3^+^ cells (red, top) in kidneys of 12-, 20- and 40-week-old *Ctns* rats (*n* = 67–95 areas pooled from three rats per group). (**B**) Representative micrographs of Picro-Sirius Red staining and quantification of fibrotic tissue in the cortex relative to tissue surface (bottom, *n* = 5 *Ctns*^+/+^ and *n* = 8 *Ctns*^−/−^ rats per group). The black dotted line delineates the cortex from the medulla. Insets: high magnification of the corresponding section. (**C**) RT-qPCR analysis of inflammatory markers *Cd3g, Ccl19, Lgals3, Tlr4* and fibrotic markers *Col6a1, Col1a1, Fn1, Col3a1, Vim* in kidneys of 20-week-old rats. Gene target expression normalized to *Gapdh* and relative to *Ctns*^+/+^ rats (black dotted line; *n* = 12 *Ctns*^+/+^ rats and *n* = 15 *Ctns*^−/−^ per group). (**D**) Representative picture of swan-neck lesion (dotted line) and confocal micrographs of cleaved Caspase 3 (Casp 3, red). (**E**) RT-qPCR analysis of *Lcn2*, *Havcr1*, *Cts3*, *Clu* and *Ccn1* expression in *Ctns* rat kidneys. Gene target expression normalized to *Gapdh* and relative to *Ctns*^+/+^ rats (black dotted line; *n* = 6 rats per group). (**F** and **G**) Biochemical analysis of (F) blood urea nitrogen (BUN) levels and (G) the enzymatic creatinine levels measured from *Ctns* rat plasma samples at different ages (*n* = 10 rats per group). (**H**) Percentage survival of male and female *Ctns* rats over time (at day 0: *n* = 17 male *Ctns*^+/+^, *n* = 19 male *Ctns*^−/−^, *n* = 19 female *Ctns*^+/+^ and *n* = 20 female *Ctns*^−/−^ rats; at week 96: *n* = 7 male *Ctns*^+/+^, *n* = 0 male *Ctns*^−/−^, *n* = 8 female *Ctns*^+/+^ and *n* = 0 female *Ctns*^−/−^ rats). Proximal tubules labeled by AQP1 (green) and nuclei counterstained with DAPI (gray) in (A and D). Scale bars: 20 μm in (A), 500 μm in (B) and 50 μm in (D). Plotted data represent mean ± SEM. Two-tailed unpaired Student’s *t*-test, ^*^P < 0.05, ^*^^*^P < 0.01, ^*^^*^^*^P < 0.001 and ^#^*P <* 0.0001 relative to *Ctns*^+/+^ rats. ns: not significant, G: glomerulus.

We performed histological analyses to better characterize kidney disease progression in *Ctns*^−/−^ rats. Interstitial inflammatory cell infiltrates were first observed at 12 weeks, increasing over time (Fig. 3A). As inflammation is the initial response to kidney injury, promoting fibrosis (32), we detected a strong increase of expression of the profibrotic factor galectin-3 (33) also from 12 weeks of age (Supplementary Material, Fig. S2A). Picro-Sirius red staining revealed progressive kidney fibrosis in *Ctns*^−/−^ rats from 20 weeks of age onwards, starting in the cortical regions and progressively extending to the outer and inner parts of the medulla (Fig. 3B; Supplementary Material, Fig. S2B). These changes were associated with the strong upregulation of genes involved in inflammation (e.g. *Cdc3g*, *Ccl19*, *Lgals3*, *Tlr4*) and fibrosis (e.g. *Col6a1*, *Col1a1*, *Fn1*, *Col3a1*, *Vim*) in *Ctns*^−/−^ kidneys (Fig. 3C).

Tubular lesions started at 20 weeks in *Ctns*^−/−^ rats, with a flattening of the PT cells starting at the glomerulotubular junction, characteristic for the ‘swan-neck’ deformities observed in cystinosis patients and associated with apoptosis, monitored by cleaved caspase3 (Casp3) staining (Fig. 3D) and expression (Supplementary Material, Fig. S2D and E) in *Ctns*^−/−^ compared with *Ctns*^+/+^ kidneys. Of note, we did not detect apoptosis in glomerular cells at the investigated time points (Supplementary Material, Fig. S2E). Additional structural damages observed in *Ctns*^−/−^ rats included dilated renal corpuscles with enlarged Bowman’s space, sclerotic glomeruli and protein casts in PT lumen (Supplementary Material, Fig. S2B). The presence of damaged glomeruli was illustrated by the loss of immunoglobulin G (IgG) in the urine of 40-week-old rats (Supplementary Material, Fig. S2F). In line with the tubular damage, the *Ctns*^−/−^ rats showed increased urinary levels of lipocalin-2 (LCN2) and kidney injury molecule-1 (Kim-1), starting at 3 months of age and increasing over time (Supplementary Material, Fig. S2C). These changes were paralleled by increased mRNA levels of both *Havcr1* (corresponding to Kim-1) and *Lcn2*, and additional tubular damage markers including cystatin C (*Cst3*), clusterin (*Clu*) and cysteine-rich protein (*Ccn1*) in the *Ctns*^−/−^ kidneys (Fig. 3E). The progression of structural damages was associated with kidney failure, as evidenced by an increase of blood urea nitrogen (BUN) and creatinine (Fig. 3F and G), correlating with a decreased survival in *Ctns*^−/−^ rats (Fig. 3H).

### Loss of cystinosin causes defective receptor-mediated and fluid-phase endocytosis

**Figure 4:**
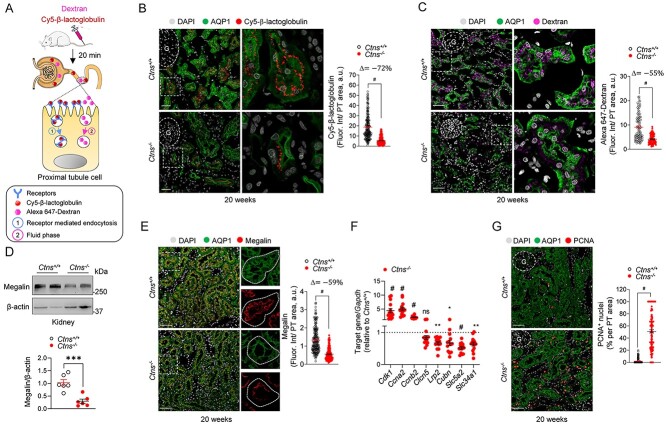
Defective receptor-mediated endocytosis and cell proliferation in *Ctns*^−/−^ rats. (**A**) Workflow of the strategy used to investigate (1) receptor-mediated endocytosis (β-lactoglobulin) and (2) fluid-phase endocytosis (dextran). After 20 min from tail vein injection with either Cy5-β-lactoglobulin (0.4 mg kg^−1^) or Alexa 647-dextran (0.2 mg kg^−1^), kidneys were fixed, processed and analyzed by confocal microscopy. (**B** and **C**) Representative confocal micrographs and quantification of the mean fluorescence intensity of (B) Cy5-β-lactoglobulin (*n* = 229 *Ctns*^+/+^ and *n* = 304 *Ctns*^−/−^ tubules, pooled from two rats per group) or (C) Alexa 647-dextran (*n* = 93 *Ctns*^+/+^ and *n* = 79 *Ctns*^−/−^ tubules, pooled from two rats per group) in AQP1^+^ (green) proximal tubules of *Ctns* rat kidneys. Insets: high magnification of Alexa 647 dextran^+^ or Cy5-labeled β-lactoglobulin^+^ structures in AQP1^+^ PTs. (**D**) Immunoblotting and quantification of megalin protein levels in whole-kidney lysates from *Ctns* rats (*n* = 6 rats per group). β-Actin was used as a loading control. (**E**) Representative confocal micrographs and quantifications of the mean fluorescence intensity of megalin (red) in AQP1^+^ (green) proximal tubules of *Ctns* rat kidneys (*n* = 249 *Ctns*^+/+^ and *n* = 280 *Ctns*^−/−^ tubules, pooled from four rats per group). Insets: high magnification of megalin^+^ structures in AQP1^+^ PTs. (**F**) RT-qPCR analysis of *Cdk10, Ccna2, Ccnb2, Clcn5, Lrp2, Cubn, Slc5a2* and *Slc34a1*. Gene target expression normalized to *Gapdh* and relative to *Ctns*^+/+^ rats (black dotted line; *n* = 9 rats per group). (**G**) Representative confocal micrographs and quantification of the percentage of PCNA^+^ (red) nuclei in AQP1^+^ (green) proximal tubules of *Ctns* rat kidneys (*n* = 150 tubules, pooled from 3 rats per group). Nuclei counterstained with DAPI (gray) in (B, C, E and G). Scale bars: 40 μm in (B, C, E and G). Fluorescence intensity was normalized on tubule area in (B, C and E). Plotted data represent mean ± SEM. Two-tailed unpaired Student’s *t*-test, ^*^P < 0.05, ^*^^*^P < 0.01, ^*^^*^^*^P < 0.001 and #*P* < 0.0001 relative to *Ctns*^+/+^ rats. G: glomerulus.

To investigate the mechanism underlying LMW proteinuria in *Ctns* rats, we monitored receptor-mediated and fluid-phase endocytosis using the *in vivo* uptake of Cy5-labeled LMW protein β-lactoglobulin and Alexa 647-dextran in the kidney, respectively (Fig. 4A). Twenty minutes after injection, a substantial accumulation of Cy5-positive vesicles was detected in the brush border/subapical region of PT cells from *Ctns*^+/+^ rats, contrasting with a major reduction of uptake in PTs from *Ctns*^−/−^ rats (Fig. 4B). Fluid-phase endocytosis was also altered in *Ctns*^−/−^ rats, as indicated by the reduced signal for Alexa 647-dextran (Fig. 4C). The defective uptake of LMW proteins was associated with a strongly decreased expression of the endocytic receptor megalin (encoded by *Lrp2*) in PTs of *Ctns*^−/−^ kidneys, both at the protein and mRNA levels (Fig. 4D–F). Of note, the mRNA levels of the co-receptor cubilin (*Cubn*), the phosphate cotransporter IIa (NaPi-IIa, *Slc34a1*) and the sodium–glucose cotransporter 2 (SGLT2, *Slc5a2*) were also decreased, in line with the LMW proteinuria, phosphaturia and glycosuria observed in *Ctns*^−/−^ animals (Fig. 4F). In parallel, *Ctns*^−/−^ rats displayed a major increase in the level of genes regulating cell cycle and driving proliferation (e.g. *Cdk1*, *Ccna2*, *Ccnb2*) compared with *Ctns*^+/+^ animals (Fig. 4F). These results were supported by a nuclear enrichment of PCNA (Fig. 4G; Supplementary Material, Fig. S3A) and Ki-67 (Supplementary Material, Fig. S3B) in PT cells of *Ctns*^−/−^ rats. The growth alterations in *Ctns*^−/−^ rats were substantiated by an increased kidney weight to body weight ratio starting at 20 weeks of age (Supplementary Material, Fig. S3C). These data indicate that cystinosin deletion in rats induced a phenotype switch associating abnormal proliferation and dedifferentiation, leading to defective endocytosis and urinary loss of solutes.

### Defective lysosomal homeostasis disrupts autophagy in *Ctns*^−/−^ kidneys

**Figure 5:**
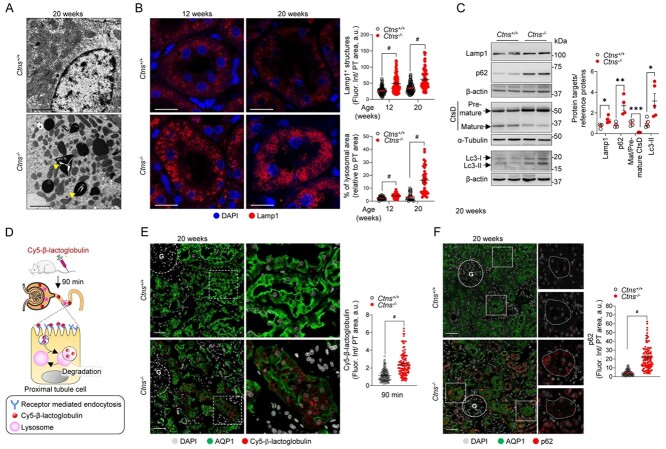
Cystine crystals and enlarged lysosomes in proximal tubules of cystinotic rats. (**A**) Representative electron micrographs of proximal tubules derived from 20-week-old *Ctns* rat kidneys. Arrowheads indicate the presence of needle-shaped crystals in dense bodies in *Ctns*^−/−^ samples. (**B**) Representative confocal micrographs of Lamp1^+^ structures (red) in proximal tubules of 12- and 20-week-old *Ctns* rats. Quantification of the mean fluorescent intensity of Lamp1 (top, each dot represents the mean fluorescent intensity per tubule, *n* = 90 tubules per condition pooled from three rats per group) and total lysosomal area (bottom, each dot represents the average size of Lamp1^+^ vesicles in one tubule, *n* = 56 *Ctns*^+/+^*n* = 60 *Ctns*^−/−^ tubules at 12 weeks, and *n* = 31 *Ctns*^+/+^*n* = 38 *Ctns*^−/−^ tubules at 20 weeks, pooled from three rats per group). Fluorescence intensity and lysosomal area were normalized on tubule area. (**C**) Western blotting and densitometry analyses of lysosomal and autophagy protein levels in whole-kidney lysates from *Ctns* rats (*n* = 4 rats per group). (**D**) Workflow of the strategy used to investigate lysosomal degradative capacity. After 90 min from tail vein injection of Cy5-β-lactoglobulin (0.4 mg kg^−1^), the labeled protein is internalized and degraded by endolysosomes. The kidneys were then processed and analyzed by confocal microscopy. (**E** and **F**) Representative confocal micrographs and quantifications of the mean fluorescence intensity of (E) Cy5-β-lactoglobulin (red; *n* = 110 *Ctns*^+/+^ and *n* = 118 *Ctns*^−/−^ tubules, pooled from two rats per group) or (F) p62 (red; *n* = 90 *Ctns*^+/+^ and *n* = 116 *Ctns*^−/−^ tubules, pooled from two rats per group) in AQP1^+^ (green) proximal tubules of *Ctns* rat kidneys. Fluorescence intensity was normalized on tubule area. Nuclei counterstained with DAPI (gray or blue) in (B, E and F). Each dot represents fluorescence intensity in one tubule in (B, E and F) or one rat (C). β-actin or α-tubulin was used as loading control. Scale bars: 2 μm in (A), 20 μm in (B), 50 μm in (E) and 40 μm in (F). Plotted data represent mean ± SEM. Two-tailed unpaired Student’s *t*-test, ^*^P < 0.05, ^*^^*^P < 0.01, ^*^^*^^*^P < 0.001 and #*P* < 0.0001 relative to *Ctns*^+/+^ rats. ns: not significant. G: glomerulus.

With cystinosin being a lysosomal membrane transporter, we next examined lysosomal dynamics in *Ctns*^−/−^ rats. Electron microscopy (EM) revealed accumulation of large and amorphous vacuoles in the kidneys of *Ctns*^−/−^ rats at 12 and 20 weeks of age, with formation of thin needle-shaped crystals within enlarged and dense vesicles in PT cells (Fig. 5A; Supplementary Material, Fig. S4A). Crystal formation was also detected in liver and thyroid samples (Supplementary Material, Fig. S4B).

The accumulation of enlarged lysosomal vesicles was substantiated by the number of Lamp1-positive vesicles, with increasing size over time, in the PT cells of *Ctns*^−/−^ kidneys (Fig. 5B). As changes in lysosomal dynamics could affect their proteolytic capacity, we analyzed whether *Ctns* deletion impairs the lysosomal cargo processing. Western blot analysis revealed a defective proteolytic generation of the 32 kDa mature form of the lysosomal enzyme cathepsin D (CtsD) (Fig. 5C), paralleled by a dramatic reduction in lysosomal processing as indicated by the persistent signal of Cy5-β-lactoglobulin in PT cells 90 minutes after injection in *Ctns*^−/−^ rats, compared with the complete processing observed in *Ctns*^+/+^ PT cells (Fig. 5D and E). As lysosomal function is crucial for maintaining autophagy, we analyzed autophagy markers in *Ctns* rat kidneys. Larger numbers of aggregates positive for the autophagy receptor Sqstm1/p62 along with higher protein levels of lipidated, autophagosome-associated form Lc3-II and Sqstm1/p62 confirmed marked alterations in autophagy process in the *Ctns*^−/−^ compared with the *Ctns*^+/+^ kidneys (Fig. 5C and F). Collectively these data indicate that the deletion of *Ctns* in rats leads to crystal formation in multiple tissues due to cystine overload. These changes are associated with impaired lysosomal degradative capacity and accumulation of autophagic cargoes.

### Defective endolysosomal function in primary proximal tubule cells from *Ctns*^−/−^ kidneys

**Figure 6:**
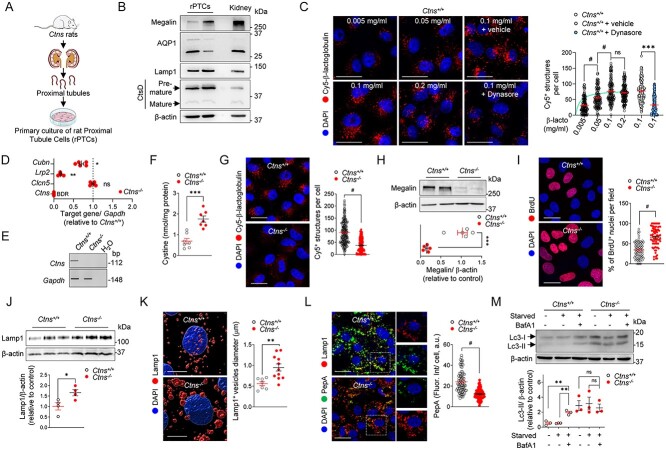
Proximal tubule cells derived from *Ctns*^−/−^ rat kidneys recapitulate key features of cystinosis. (**A**) Workflow of the strategy used to develop primary proximal tubule cells derived from *Ctns* rat kidneys (rPTCs). (**B**) Immunoblotting of proximal tubule and lysosomal markers in rPTCs. (**C**) *Ctns*^+/+^ rPTCs were loaded with Cy5-β-lactoglobulin (red; at the indicated concentration) for 20 min at 37°C or loaded with Cy5-β-lactoglobulin (red, 100 μg ml^−1^) in the presence or absence of Dynasore (40 μm) for 20 min at 37°C and analyzed by confocal microscopy. Quantification of the number of Cy5-β-lactoglobulin^+^ structures (*n* = 163–193 cells pooled from two biologically independent experiments). Each dot represents the number of Cy5-β-lactoglobulin^+^ structures in one cell. (**D**) mRNA levels of *Ctns*, *Clcn5, Lrp2* and *Cubn* in rPTC analyzed by RT-qPCR. Gene target expression normalized to *Gapdh* and relative to *Ctns*^+/+^ rPTC (black dotted line; *n* = 5 biologically independent experiments). (**E**) *Ctns* genomic DNA analyses by PCR and agarose gel electrophoresis isolated from rPTCs. (**F**) Intracellular cystine levels were measured by HPLC (*n* = 7 biologically independent experiments per group). (**G**) *Ctns*^+/+^ and *Ctns*^−/−^ rPTCs were loaded with Cy5-β-lactoglobulin (red, 100 μg ml^−1^) for 20 min at 37°C and analyzed by confocal microscopy. Quantification of the number of Cy5-β-lactoglobulin^+^ structures (*n* = 208 *Ctns*^+/+^ and *n* = 228 *Ctns*^−/−^ cells pooled from three biologically independent experiments). Each dot represents the number of Cy5-β-lactoglobulin^+^ structures in one cell. (**H**) Immunoblotting and quantification of megalin protein levels in rPTC lysates (*n* = 5 independent experiments). (**I**) Cells were loaded with bromodeoxyuridine (BrdU; 1.5 μg ml^−1^ for 16 h at 37°C), analyzed by confocal microscopy and quantified as percentage of BrdU^+^ cells per field (*n* = 77 *Ctns*^+/+^ and *n* = 79 *Ctns*^−/−^ fields containing ~30 cells, pooled from two independent experiments). (**J**) Immunoblotting and quantification of Lamp1 protein levels in rPTC lysates (*n* = 4 independent experiments per group). (**K**) High-magnification representative 3D surface renderings of *Ctns* rPTCs labeled with anti-Lamp1 (red) antibody and quantification of lysosomal vesicle diameter (μm). Each dot represents the average size of Lamp1^+^ vesicles in one cell (*n* = 6 *Ctns*^+/+^ and *n* = 11 *Ctns*^−/−^ fields). (**L**) Cells were loaded with Bodipy-FL-PepA (1 μm, green) for 1 h at 37°C, fixed, immunostained with anti-Lamp1 antibody (red) and analyzed by confocal microscopy. Quantification of PepA fluorescent signal as mean fluorescence intensity per cell (*n* = 84 *Ctns*^+/+^ and *n* = 88 *Ctns*^−/−^ cells pooled from two independent experiments). (**M**) Cells were cultured under normal and growth factors/nutrient-depleted conditions (Starved) in the presence or absence of 250 nm Bafilomycin (BafA1) for 4 h. Immunoblotting and quantification of Lc3 protein levels in rPTC lysates (*n* = 3 independent experiments). One-way ANOVA followed by Dunnet’s post hoc test, ^*^^*^P < 0.01 relative to rPTCs treated with BafA1. β-Actin was used as loading control in (B, H, J and M). Nuclei counterstained with DAPI (blue) in (C, G, I, K and L). Scale bars: 20 μm in (C, G, I and L) and 7 μm in (K). Plotted data represent mean ± SEM. Two-tailed unpaired Student’s *t*-test. ^*^P < 0.05, ^*^^*^P < 0.01, ^*^^*^^*^P < 0.001, and *#P* < 0.0001 relative to *Ctns*^+/+^ rPTCs. ns: not significant.

The primary cultured PT cells obtained from mouse kidneys (mPTCs) have been shown to keep their differentiation and polarized transport processes, representing a reliable cell system to investigate endolysosomal disorders in particular (9,34–37). To further investigate the mechanism of cystinosis *in vitro*, we established primary cultures of proximal tubule cells (rPTCs) derived from micro-dissected proximal tubules of *Ctns* rat kidneys (Fig. 6A; Supplementary Material, Fig. S5A and B). The rPTCs display high levels of PT (e.g. megalin and AQP1), endolysosome (e.g. Rab 5, Rab 7 Rab 11, Lamp1 and CtsD) and autophagy (e.g. Lc3 and p62) markers, without expressing markers of other kidney segments (e.g. *Aqp2*: collecting duct, *Umod:* thick ascending limb, *Npsh2:* glomerulus) (Fig. 6B; Supplementary Material, Fig. S5C and D). The rPTCs exhibit a high and saturable endocytic capacity, which is reduced by Dynasore (40 μm for 1 h), an inhibitor of dynamin involved in membrane fusion during endocytosis (Fig. 6C), and are polarized, as shown by the apical and basolateral expression of megalin and Na^+^, K^+^-ATPase, respectively (Supplementary Material, Fig. S5E).

As a cystinosis disease model, the rPTCs from *Ctns*^−/−^ kidneys showed absence of *Ctns* mRNA expression (Fig. 6D and E) and increased cystine content (Fig. 6F). The accumulation of cystine in *Ctns*^−/−^ rPTCs induced a phenotype switch similar to that observed *in vivo,* with apical dedifferentiation leading to defective receptor-mediated endocytosis (Fig. 6G and H), abnormal proliferation (Fig. 6I) and dramatic alterations in lysosomal homeostasis with increased Lamp1 and enlarged lysosomes (Fig. 6J and K). These changes led to impaired lysosomal proteolytic activity, illustrated by the reduced number of PepstatinA-positive lysosomes, a bona fide biosensor (11) that binds to the active site of CtsD in acidic lysosomes (Fig. 6L).

We next verified whether the defective lysosomal homeostasis had an impact on autophagy in *Ctns*^−/−^ cells. Autophagy was assessed by quantifying the conversion of the non-lipidated form of Lc3-I to the lipidated, autophagosome-associated form Lc3-II in rPTCs cultured in nutrient-rich media (hereafter referred to as ‘fed’) or in nutrient-deprived conditions (hereafter referred to as ‘starved’). Compared with wild-type *Ctns*^+/+^ cells, *Ctns*^−/−^ cells showed higher levels of Lc3-II, which did not further increase under starved conditions. Treatment with Bafilomycin A1 (BafA1) blocks lysosome acidification and thus increases accumulation of autophagic cargo in *Ctns*^+/+^ cells. However, no further increase of the already elevated levels of Lc3-II in nutrient-deprived *Ctns*^−/−^ cells was detected (Fig. 6M). These results demonstrate that the deletion of cystinosin alters lysosomal dynamics and autophagy in rPT cells. These data establish rPTCs as a well-differentiated primary cell culture system matching key features of nephropathic cystinosis.

### Ocular manifestations, bone and liver defects in the *Ctns*^−/−^ rat model

**Figure 7:**
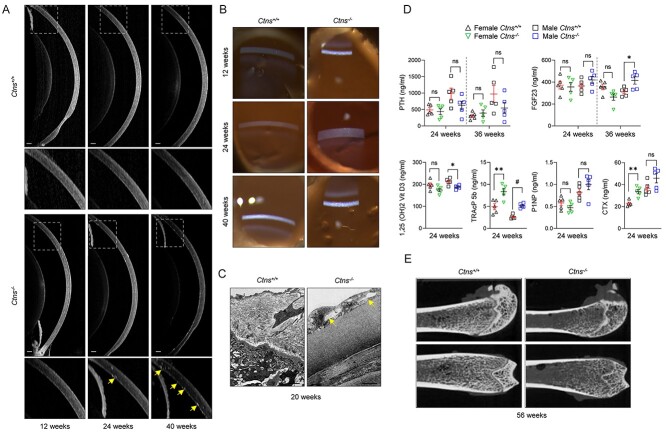
*Ctns* deletion causes corneal crystal formation and bone defects *Ctns*^−/−^ rats. (**A**) Anterior eye OCT imaging of *Ctns* rat eyes at 12, 24 and 40 weeks of age. Insets: high magnification of indicated areas (yellow arrows: crystals). (**B**) Slit-lamp photography of *Ctns* rat eyes at 12, 24 and 40 weeks of age. (**C**) Representative electron micrographs of 20-week-old *Ctns* rat eyes (yellow arrows: crystals). (**D**) Parathyroid hormone (PTH), fibroblast growth factor 23 (FGF23), 1-a hydroxylation of vitamin D3 (1,25(OH)2 Vit D3), tartrate-resistant acid phosphatase (TRAcP 5b), C-terminal telopeptide of type I collagen (CTX I) and N-terminal propeptide of type I procollagen (P1NP) levels in plasma from 24- and 36-week-old *Ctns* rats. (**E**) Representative microCT images of femurs derived from *Ctns* rats at 56 weeks of age. Plotted data represent mean ± SEM. Scale bars: 200 μm in (A), 2 μm in (C). Two-tailed unpaired Student’s *t*-test. ^*^P < 0.05, and ^*^^*^P < 0.01, relative to *Ctns*^+/+^ rPTCs. ns: not significant.

We finally examined whether *Ctns*^−/−^ rats presented extra-renal manifestations, as frequently observed in patients with cystinosis. Potential crystal formation was investigated in the cornea, as cystine overload was detected in the eyes of *Ctns*^−/−^ rats (Fig. 1D). At 12 weeks of age, *Ctns*^+/+^ and *Ctns*^−/−^ animals were indistinguishable from each other (Fig. 7A). At 24 weeks, white, hyper-reflective and needle-shaped deposits were detectable in the eyes of *Ctns*^−/−^ rats by optical coherence tomography (OCT). In 40-week-old animals, crystals became larger and more abundant. Slit-lamp imaging revealed a broad distribution of crystals in the corneas of both eyes of the examined animals (Fig. 7A and B), and EM confirmed the deposition of crystals in the corneal endothelium of *Ctns*^−/−^ rats (Fig. 7C). Slit-lamp imaging also revealed the presence of corneal dellen in both *Ctns*^+/+^ and *Ctns*^−/−^ rats (Fig. 7B).

High levels of cystine (Fig. 1D) and cystine crystals (Supplementary Material, Fig. S4B) were detected in the liver of *Ctns*^−/−^ rats at 20 weeks. Analysis of hepatotoxicity markers including aspartate-aminotransferase (AST), alanine-aminotransferase (ALT), alkaline phosphatase (ALP) and gamma-glutamyl transferase (GGT) revealed a significant increase of both ALT and GGT in the plasma of 20 weeks in *Ctns*^−/−^ rats (Supplementary Material, Fig. S6 and Table S2), supporting the development of liver damage.

Cystinosin deficiency was also reflected on the bone physiology of *Ctns*^−/−^ rats. The levels of parathyroid hormone (PTH) and fibroblast growth factor 23 (FGF23) were similar between *Ctns*^+/+^ and *Ctns*^−/−^ rats at 24 weeks, while 1,25-dihydroxyvitamin D (1,25(OH)2 Vit D3) levels were significantly decreased in *Ctns*^−/−^ rats, likely reflecting PT dysfunction (Fig. 7D and Supplementary Material, Table S3). At 36 weeks, increased FGF23 levels were observed in male *Ctns*^−/−^ rats, in line with defective phosphate homeostasis. The levels of tartrate-resistant acid phosphatase (TRAcP 5b, an osteoclast marker) and C-terminal telopeptide of type I collagen (CTX I, a bone resorption marker) were increased in *Ctns*^−/−^ rats (Fig. 7D and Supplementary Material, Table S3), indicating the development of osteoporosis. No differences were observed in the level of the bone formation marker N-terminal propeptide of type I procollagen (P1NP). Micro-computed tomography (Micro-CT) performed on 56-week-old animals showed a significant decrease in bone volume/total volume, cortical bone area, trabecular number and an increase in trabecular separation in *Ctns*^−/−^ rats (Fig. 7E; Supplementary Material, Table S4). Together, these modifications suggest the development of eye and liver defects, and inadequate bone mineralization and rickets in *Ctns*^−/−^ rats, similar to patients with cystinosis.

## Discussion

Animal models are crucial to gain mechanistic insights and to accelerate drug development in rare genetic disorders. Triggered by the shortcomings of the mouse model of cystinosis, we describe the first *Ctns*-deficient rat generated by CRISPR/Cas9. The *Ctns*^−/−^ rat model shows a major accumulation of cystine in all tissues, causing typical kidney damage and multisystem complications encountered in patients with cystinosis. The *Ctns*^−/−^ rats show a progressive, generalized dysfunction of the proximal tubule, complicated by kidney failure and a shorter life expectancy. Mechanistically, the accumulation of cystine is causing a switch of the phenotype of proximal tubule cells, with increased proliferation and loss of apical receptors and transporters, driving the urinary loss of LMW proteins and solutes. The cystine storage triggers severe alterations in lysosomal homeostasis, with defective proteolysis and accumulation of autophagic cargos in the cells.

The *Ctns*^−/−^ rat model recapitulates the early kidney tubular and structural defects, leading to generalized PT dysfunction and kidney failure, similar to patients with nephropathic cystinosis (3). These key features are not observed in the LEA/Tohm rat carrying a spontaneous 13 bp-deletion in the *Ctns* gene with moderate accumulation of cystine in some tissues (23). These rats show isolated glycosuria (hence named *Ctns^ugl^*), with no other manifestation of PT dysfunction, no kidney failure, no growth defect and no other complications observed in cystinosis (23). The discrepancy between both models is presumably due to the use of CRISPR/Cas9, permitting specific and efficient disruption of *Ctns* in our model, compared with the spontaneous mutation in the *Ctns^ugl^* line. The distinct backgrounds of the rats—pure Sprague–Dawley versus LEA/Tohm backcrossed to the inbred F344 strain—may also play a role, as suggested by the strong effect of the mouse background documented for the *Ctns* mouse model (10).

The PT dysfunction observed in the *Ctns*^−/−^ rat associates defective receptor-mediated endocytosis, as observed in other endolysosomal disorders (1), and impaired fluid-phase endocytosis. These modifications reflect the abnormal expression and/or recycling of the multi-ligand receptor megalin and the severity of epithelial dysfunction in PT cells of the *Ctns*^−/−^ rat. It should be noted that a compromised fluid-phase endocytosis has been observed in a mouse model for Dent disease 1 exhibiting strong PT dysfunction (36), in a fish model for Dent disease 2 (38) and in a *lrp2* (megalin)-deficient zebrafish model (39,40). These data demonstrate the severity of PT dysfunction in *Ctns*^−/−^ rats, starting at 6 weeks of age, in contrast to the *Ctns*^−/−^ mice showing a later onset and only mild/incomplete Fanconi syndrome. Further comparative studies will need to decipher the molecular mechanisms and cellular pathways involved in these inter-species differences in PT dysfunction.

The fundamental trigger of cystinosis is the accumulation of cystine causing a lysosomal storage disease (41). Cystine crystals were observed in *Ctns*^−/−^ rats at 12 weeks, earlier than the reported 24 weeks in *Ctns*^−/−^ mice (14). The difference could be linked to cysteine levels between rats and mice, as crystals appear when the concentration of cystine exceeds 5 mm (42). In fact, *Ctns*^−/−^ rats exhibited a higher cystine concentration in kidney (~40 nmol mg^−1^ protein, 20 weeks) compared with *Ctns*^−/−^ mice (~15 nmol mg^−1^ protein, 24 weeks) (11).

Profound alterations in lysosomal dynamics, including accumulation of enlarged Lamp1-positive vesicles and large and amorphous vacuoles filled with crystals, were observed in the PT cells of *Ctns*^−/−^ kidneys. These changes are reflected by alterations in the lysosomal cargo processing and proteolytic activity, and accumulation of autophagic cargo. Accumulation of p62 and Lc3-II, two autophagy markers that are normally degraded in lysosomes, suggests an impairment of the autophagy flux, in line with previous studies on kidneys and primary PT cells derived from *Ctns*^−/−^ mice (11,12). The conjunction of lysosomal dysfunction, defective autophagy and loss of differentiation contrasting with increased markers of proliferation in the *Ctns*^−/−^ rats substantiates the concept of the lysosome being at the crossroad of regulating cellular proliferation and differentiation (11,43). At the cellular level, the association of abnormal proliferation and dedifferentiation is causing defects in the apical transport systems that operate in PT cells, explaining the renal Fanconi syndrome (11).

The limitations of immortalized cell culture systems to investigate highly differentiated epithelial cells have been emphasized (11,34,44). Primary culture systems of PT cells derived from mouse kidneys (mPTC) have been crucial to decipher mechanisms underlying endolysosomal disorders affecting the kidney (11,34,37,45). In order to validate this approach and substantiate disease mechanisms, we established a primary culture system of PT cells derived from *Ctns* rat kidneys. These rPTCs show a polarized expression of essential markers such as megalin and cubilin and a high receptor-mediated endocytic capacity. The rPTCs derived from *Ctns*^−/−^ kidneys showed critical aspects of the disease, including accumulation of cystine, impaired endocytosis, loss of differentiation and increased proliferation, and impaired lysosomal homeostasis leading to altered autophagy degradation. The use of the fluorescent biosensor PepA, which binds to the active site of CtsD in acidic lysosomes, revealed a significantly decreased fluorescent signal in *Ctns*^−/−^ rPTCs compared with *Ctns*^+/+^ cells, substantiating the impaired lysosomal degradative capacity due to defective maturation of cathepsins (46). These findings are in line with previous observations obtained in mPTCs (9,39), validating the rPTCs as a cellular disease model for cystinosis.

Due to continuous exposure to cystine storage, patients with cystinosis develop systemic manifestations later in life. By the age of 10 years, half of the patients develop extra-renal manifestations including reduced vision, liver dysfunction and impaired growth and rickets (47,48). The appearance of crystals in the corneas of *Ctns*^−/−^ rats, increasing with age, correlates with the corneal manifestations observed in patients that cause photophobia and vision deterioration. The *Ctns*^−/−^ rats also showed an accumulation of cystine in the liver, leading to the formation of crystals and elevated markers of liver dysfunction. They also presented alterations of the cortical and trabecular structures in long bones. These alterations may reflect the loss of phosphate and calcium due to PT dysfunction and reduced activation of vitamin D, reflecting the situation observed in patients (49). A contribution of reduced bone remodeling activity, which may be due to a defect in osteoblasts and osteoclasts, may also contribute to the phenotype (50).

The availability of a faithful rat model, with inherent advantages for genetic conservation, pharmacology and toxicology, and relevance for metabolism and kidney pathophysiology (51), is an important step for translational research in cystinosis. The *Ctns*^−/−^ rats develop a set of kidney and extra-renal manifestations that recapitulate the human disease in terms of timing, severity, and histopathological changes. These changes reflect the lysosome disease at the cellular level, with defective autophagy and homeostatic processes that could offer therapeutic targets (8,9,11). Studies taking advantage of the rat model, combined with other model organisms, will further decipher these mechanistic links and accelerate the translation of novel therapeutic strategies for cystinosis patients.

In summary, this novel *Ctns*^−/−^ rat model of cystinosis recapitulates essential clinical and molecular features of cystinosis, including the role of defective endolysosomal dynamics and autophagy. This rat model and the derived cell culture system represent powerful tools to accelerate translational research in cystinosis.

## Materials and Methods

### Generation and maintenance of the *Ctns* rat model

The CRISPR/Cas9 system was used to delete the *Ctns* gene in Sprague–Dawley rats (PolyGene AG, Zurich, Switzerland). Two single-guide RNAs (sgRNAs) targeting exon 3 of *Ctns* were selected: CRISPR1a: ACCAACGTCAGCATTACCCT(TGG), CRISPR1b:CCATTTACCAGCTTCACAGT(GGG). Before injection, the sgRNA sequences were blasted against the rat genome for off-targets. A total of 146 rat embryos were injected with the combination of CRISPRs 1a/b. From these embryos, 90 survived and could be transferred into foster rats. A total of 34 pups were born from these injections, which were analyzed for changes in the *Ctns* locus. PCR was used to amplify the *Ctns* region surrounding the CRISPR-target site using the following primer combination: CTNS_R: 5′-ACACCCGAAGTACATGCAGA-3′, CTNS_L:5′-ACAGAGATGGG AAGAGCACA-3′. The resulting PCR product was digested by T7 endonuclease. Five animals showed a positive signal indicating insertion or deletion in the *Ctns* locus. These changes were analyzed via PCR and sequencing. The following primer combinations were used 5′-AGGCACGATGGAGCAGTAAAG-3′ and 5′-ATGCACGAATGAGACCAGACC-3′. A *Ctns* rat line harboring a deletion of 12 bp and insertion of 8 bp resulting in a premature stop codon in the exon 3 of *Ctns* gene was selected (Fig. 1). Potential off-target sites were identified using an *in silico* tool, Cas-OFFinder (31).

All experiments were performed on male and female animals, unless specified, and were conducted on age- and gender-matched *Ctns*^−/−^ and *Ctns*^+/+^ rat littermates. Rats were maintained under temperature- and humidity-controlled conditions with 12 h light/12 h dark cycle with free access to appropriate standard diet in accordance with the institutional guidelines of National Institutes of Health Guide for the Care and Use of Laboratory Animals. Kidney and other tissues were collected for analyses at the time of sacrifice. The experimental protocols were approved by the appropriate licensing committee (Kanton Zürich Gesundheitsdirektion Veterinäramt; protocol ZH0230/2019) at the University of Zurich.

### Genotyping

Genomic DNA was isolated from ear punch biopsies of *Ctns* rats by using E.Z.N.A Forensic DNA Kit (OMEGA bio-tek, Norcross, UK) according to the manufacturer’s instructions. The *Ctns* genotyping was performed using the primers 5′-GGACCCATTCACTGTCCATC-3′ (forward) and 5′-GACATGTGGGACCCTTTGAT-3′ (reverse). The nucleotide deletion/insertion change was confirmed by Sanger sequencing, using an Applied Biosystems 3730 DNA Analyzer (Applied Biosystems, Foster City, CA, USA).

### Kidney function parameters

Rats were placed for 16 h in metabolic cages with ad libitum access to food and drinking water. The body weight, water intake and urinary volume were measured, and urine was collected over ice. Urine and blood parameters were measured by using a UniCel DxC 800 Pro Synchron (Beckman Coulter, Fullerton, CA, USA). The concentration of the LMW Clara cell protein (CC16) in urine was measured in duplicate by enzyme-linked immunosorbent assay (ELISA; abx155347, ABEXA, Cambridge, UK). Albuminuria was measured via Coomassie Blue staining by using ProtoBlue Safe (EC-722, National Diagnostics, Atlanta, GA, USA) according to the manufacturer’s instructions.

### Cystine measurement

Tissue samples from rats or primary cultured cells were homogenized and lysed with N-ethylmaleimide (NEM) solution containing 5.2 mmol l^−1^*N*-ethylmaleimide in 10 mmol l^−1^ potassium phosphate buffer adjusted to pH 7.4. The lysates were collected and precipitated with sulfosalicylic acid (12% w/v) and centrifuged at 10 000 r.p.m. for 10 min at 4°C. The resulting supernatant was dissolved in citrate loading buffer (Biochrom Ltd, Cambridge, UK) and 50 μl of this solution was analyzed by Biochrom 30 Plus Amino Acid Analyzer (Biochrom Ltd). The protein pellet was dissolved in 0.1 mol l^−1^ NaOH solution and the protein concentration was determined by the Biuret method. The concentration of amino acids was measured by using a lithium high-performance physiological column (Biochrom Ltd) followed by postcolumn derivatization with ninhydrin. The amino acids were identified according to the retention time and the ratio of the area between the two wavelengths (570 and 440 nm) and quantified by using EZChrom Elite software (Agilent Technologies Inc., Pleasanton, CA, USA). Cystine concentration was normalized to protein concentration and reported in nmol per mg protein (11).

### Kidney sample processing

Rats were anesthetized in accordance with the institutional guidelines of National Institutes of Health Guide for the Care and Use of Laboratory Animals, by intraperitoneal injection with a combination of ketamine (100 mg ml^−1^; Streuli Pharma AG, Uznach, Switzerland) and xylazine (20 mg ml^−1^; Streuli Pharma AG). At time of sampling, one kidney was clamped, split transversally and one half was flash frozen in liquid nitrogen, homogenized by Dounce homogenizer in 1 ml of RIPA buffer that contained phosphatase and protease inhibitors and processed for western blot analysis. The other half was flash frozen in liquid nitrogen and used for RT-qPCR analysis. The contralateral kidney was perfused with PBS, followed by 50–60 ml 4% PFA solution in PBS (158127, Sigma-Aldrich, St.-Louis, MO, USA). The kidney was then fixed and processed for immunostaining.

### Histological studies

Kidneys from *Ctns* rats were isolated and fixed in 4% PFA as described above. Following dehydration, kidneys were embedded in paraffin, and paraffin blocks were sectioned into 5-μm-thick slices with a Leica RM2255 rotary microtome (Thermo Fisher Scientific, Waltham, MA, USA) on Superfrost Plus glass slides (Thermo Fisher Scientific). Before staining, slides were deparaffinized in Xylenes (534056, Sigma-Aldrich) and rehydrated. Picro-Sirius Red Solution (ab150681, Abcam, Cambridge, UK) was used according to the manufacturer’s protocol. The slides were mounted in Toluene mounting medium (SP15-500, Fisher Scientific, Hampton, NH, USA) reagent and acquired on an automated Zeiss Axio Scan.Z1 slidescanner (Center for Microscopy and Image Analysis, University of Zurich), equipped with a Plan Apochromat × 40 NA 0.95 air-immersion objective. Quantitative analysis was performed by color deconvolution of collagen and non-collagen components (stained red and orange, respectively), via ImageJ software and the quantification of fibrotic tissue relative to total tissue surface (52).

### Immunofluorescence

Kidneys from *Ctns* rats were isolated and fixed in 4% PFA as described above. After fixation in 4% PFA overnight at 4°C, tissue was either snap-frozen in cryogenic Tissue-Tek OCT compound (Electron Microscopy Sciences, Hatfield, PA, USA) or dehydrated and embedded in paraffin at 58°C. The embedded kidneys were sectioned at 5 μm with either a Leica cryostat (Leica Biosystems, Wetzlar, Germany) or a Leica RM2255 rotary microtome (Thermo Fisher Scientific) on Superfrost Plus glass slides (Thermo Fisher Scientific). Paraffin-embedded kidney sections were deparaffinized in changes of Xylene (534056, Sigma-Aldrich). Antigen retrieval was accomplished by incubating in sodium citrate buffer (1.8% 0.1 M citric acid, 8.2% 0.1 M sodium citrate, in distillated water of pH 6.0) in a microwave histoprocessor, HistosPRO (SW 2.0.0, Milestone, Brondby, Denmark) for 10 min at 95°C. After rehydration, paraffin- and cryo-sections were blocked for 30 min with blocking buffer (5% BSA in PBS Ca/Mg (D1283, Sigma–Aldrich) and incubated overnight at 4°C with primary antibodies. After three PBS rinses, the slides were incubated with the corresponding fluorophore-conjugated Alexa secondary antibodies (Invitrogen, Waltham, MA, USA) diluted in blocking buffer at room temperature for 1 h. Nuclei were counterstained with 1 μm 4′,6-diamino-2-phenylindole dihydrochloride (DAPI; D1306, Thermo Fischer Scientific). Slides were mounted in Prolong Gold Anti-Fade Reagent (P36930, Thermo Fisher Scientific).

For immunofluorescence analysis, rPTCs were cultivated in one-well chamber slides (81156, Ibidi, Gräfelfing, Germany) and then fixed for 10 min with 4% PFA in PBS, quenched with 50 mm NH4Cl and permeabilized for 20 min in blocking buffer solution containing 0.1% Triton X-100 and 0.5% BSA dissolved in PBS. Subsequently, rPTCs were incubated with the suitable primary antibodies overnight at 4°C. After three PBS washes, chambers were incubated for 1 h with the appropriate fluorophore-conjugated Alexa secondary antibodies (Invitrogen) and counterstained with 1 μm Dapi for 10 min. Cells were mounted in Prolong Gold Anti-Fade Reagent.

Acquisitions were performed using a confocal laser scanning microscope Leica SP8 inverse FALCON or a confocal laser scanning microscope Leica SP8 inverse (Center for Microscopy and Image Analysis, University of Zurich). Both microscopes were equipped with a Leica APO x 63 NA 1.4 oil-immersion objective. In both cases, images were acquired with a resolution of 1024 × 1024 pixels and the pinhole diameter adjusted to 1 Airy unit for each emission channel. The quantitative cell image analyses were performed by using ImageJ software and the open-source cell image analysis software CellProfiler™ (36).

### Electron microscopy

For electron microscopy, 1–2 mm fragments of tissues (12- and 20-week-old rats) were fixed in Karnovsky fixative (2.5% glutaraldehyde solution buffered at pH 7.2 + paraformaldehyde 4% solution buffered at pH 7.2, 0.13 mol l^−1^ in equal part), post-fixed in 1% osmium tetroxide for 1 h, dehydrated in graded ethanol solutions and in propylene-oxide and embedded in epoxy resin. Semithin sections from each sample were stained with Azur II-Methylene Blue, in order to select appropriated fields. Ultra-thin sections were cut with diamond knife on Ultramicrotome Reichert, placed on uncoated grids, contrasted with uranyl acetate and lead citrate, and observed with a Jem-1400 Plus Electron Microscope.

### Western blotting

Proteins were extracted from rat kidneys or rPTCs. Kidney tissues were lysed using a RIPA lysis buffer (R0278, Sigma-Aldrich) containing protease (1836153001, Roche, Basel, Switzerland) and phosphatase inhibitors (04906845001, PhosSTOP Sigma- Aldrich), and homogenized with a disperser (Z722359, IKA-Werke, Staufen im Breisgau, Germany). rPTCs were lysed using the same lysis buffer. Following sonication and centrifugation at 12000 r.p.m. for 10 min at 4°C, tissue and cell samples were thawed on ice, normalized for protein (20 μg per lane) and dissolved in Laemmli sample buffer. After separation by SDS-PAGE in reducing conditions, blotting onto polyvinylidene difluoride membranes and blocking with 5% non-fat milk (1706404, Bio-Rad, Hercules, CA, USA) diluted in PBS, the membranes were incubated overnight at 4°C with primary antibody. The next day, washing, incubation with peroxidase-labeled secondary antibody, and visualization using enhanced chemiluminescence (WBKLS0050, Merck Millipore, Burlington, MA, USA) followed. For re-probing, the membranes were rinsed and incubated for 30 min at 55°C in a stripping buffer (62.5 mmol^−1^ Tris–HCl, 2% SDS, 100 mm mercaptoethanol, adjusted to pH 7.4), before incubation with primary antibodies. Quantitative analyses were performed by scanning the blots and measuring the relative density of each band normalized to β-actin or α-tubulin with ImageJ software.

### RT-quantitative-PCR

Total RNA was extracted from whole rat kidneys using Aurum Total RNA Fatty and Fibrous Tissue Kit according to manufacturer’s protocol (Bio-Rad). To eliminate genomic DNA contamination, DNAse I treatment was performed. Total RNA was extracted from primary cell cultures with RNAqueous kit (Applied Biosystems, Life Technologies, Carlsbad, CA, USA). One microgram of RNA was used to perform the reverse transcriptase reaction with iScript cDNA Synthesis Kit (Bio-Rad). Changes in the expression level of the target genes were determined by relative reverse transcriptase-quantitative PCR with a CFX96 Real-Time PCR Detection System (Bio-Rad) using iQ SYBR Green Supermix (Bio-Rad). RT-qPCR analyses were performed in duplicate, using 100 nm of both sense and anti-sense primers in a final volume of 20 μL using iQ SYBR Green Supermix (Bio-Rad). Designing of specific primers was performed with Primer3 (Supplementary Material, Table S5). PCR conditions were 95°C for 3 min followed by 40 cycles of 15 s at 95°C, 30 s at 60°C. The PCR products were sequenced with the BigDye terminator kit (Perkin Elmer Applied Biosystems, Waltham, MA, USA) using ABI3100 capillary sequencer (Perkin Elmer Applied Biosystems). The efficiency of each set of primers was determined by dilution curves. The program geNorm version 3.4 was applied to characterize the expression stability of the candidate reference genes in kidneys, and six reference genes were selected to calculate the normalization factor. The relative changes in targeted genes over Gapdh mRNAs were calculated using the 2^−ΔΔCt^ formula.

### Primary culture of rat PT cells

The primary culture system of rat proximal tubule cells (rPTCs) was generated using a modified version of the protocol used for mouse PT cells (11). Following sacrifice and kidney isolation on ice, the cortex was dissected visually in ice cold dissection solution (DS, HBSS (14175–053, Thermo Fisher Scientific) supplemented with 10 mmol l^−1^ of glucose, 5 mmol l^−1^ glycine, 1 mmol l^−1^ alanine, 15 mmol l^−1^ HEPES (15630-056, Thermo Fisher Scientific) and 9 mmol l^−1^ mannitol pH 7.4) and sliced into pieces of ~1 mm wide. The fragments were then digested in collagenase solution (DS supplemented with 0.1% (wt/vol) type-2 collagenase (L5004177, Worthington Biochemical Corporation, Lakewood, NJ, USA) and 96 μg ml^−1^ soybean trypsin inhibitor (93620, Sigma Aldrich)) for 30 min at 37°C. Fragments were sieved through two nylon sieves (pore size 300 and 80 μm, PA-3MF-300 and PA-17-80, Sefar AG, Thal, Switzerland), where the PT fragments remained in the 80 μm sieve. The PT segments were seeded onto one-well chamber slides (81156, Ibidi) and/or collagen-coated 24-well plates (142475, Thermo Fisher Scientific) and/or collagen-coated 0.33 cm^2^ PTFE filter membranes (Corning Inc., Corning, NY, USA), and cultured at 37°C and 5% CO_2_ in Dulbecco’s modified Eagle’s medium/F12 (21041-025, Thermo Fisher Scientific) with 2.5% dialyzed fetal bovine serum (FBS), 15 mm HEPES (H0887, Sigma-Aldrich), 0.55 mm sodium pyruvate (P2256, Sigma-Aldrich), 0.1 ml l^−1^ non-essential amino acids (M7145, Sigma-Aldrich), hydrocortisone, human epidermal growth factor, epinephrine, insulin, triiodothyronine, transferrin (TF) and gentamicin/amphotericin (Single Quots kit, CC-4127, Lonza, Basel, Switzerland), pH 7.40, 325 mOsm kg^−1^. The medium was replaced every 48 h. Confluent monolayers of rPTCs were expanded from the tubular fragments after 5–7 days, characterized by a high endocytic uptake capacity. These cells were mycoplasma free. All experiments were performed on confluent monolayers grown on chamber slides or plates. The cells were derived from age- and gender-matched *Ctns*^−/−^ and *Ctns*^+/+^ rat littermates.

### Functional studies in rPT cells

#### Starvation of primary PT cells and treatments

The cells were placed in nutrient-deprived medium, after serum and amino acid removal, by washing with Hank’s balanced salt solution (55021C, Sigma-Aldrich). For uptake experiments, cells were starved for 4 h in nutrient-deprived medium. Where indicated, lysosomal proteolysis was inhibited by addition of BafA1 (250 nm for 4 h). Afterwards, the cells were processed for western blot and immunofluorescence as described above (11).

#### Endocytosis assay

The endocytic capacity of *Ctns* rat PTs *in vivo* was examined by measuring β-lactoglobulin and dextran uptake. β-Lactoglobulin was tagged with Cy5 using a TM2 Ab labeling kit (GERPN4000, GE Healthcare, Chicago, IL, USA) in accordance with the manufacturer’s instructions. Twenty minutes after tail-vein injection of Cy5-β-lactoglobulin (0.4 mg kg^−1^ B.W., L3908, Sigma-Aldrich) or 30 min after injection of 10 kDa Alexa 647-dextran (0.2 mg kg^−1^ B.W.; D22914, Thermo Fisher Scientific), rats were anesthetized and their kidneys were harvested and processed for confocal microscopy. The endocytic uptake of *Ctns* rPTCs was monitored using Cy5-β-lactoglobulin. The cells were incubated with the indicated concentrations of Cy5-β-lactoglobulin, diluted in FBS-deprived culture medium for 20 min at 37°C (36).

#### Lysosomal activity and degradation assays

*In vitro*, the lysosomal activity and degradation capacity were assessed by using Bodipy-FL-PepstatinA (P12271, Thermo Fischer Scientific) according to the manufacturer’s specifications. *Ctns* rPTCs were incubated with 1 μm Bodipy-FL-Pepstatin A in pre-warmed media at 37°C for 1 h. After washing, immunostaining with anti-Lamp1 and suitable secondary antibody was performed. Results were analyzed using confocal microscopy (11). *In vivo*, the lysosome-based processing in rats was assessed by confocal analysis of kidney PTs after 90 min from tail-vein injection of Cy5-β-lactoglobulin.

#### Cell proliferation

To assess the rate of proliferation in rPTCs, we used the Click-iT® Plus EdU Alexa Fluor® 488 Imaging Kit (C10637 Life Technologies). Cells were incubated with EdU solution for 16 h at 37°C and processed in accordance with the manufacturer’s instructions. DAPI (4′,6-diamino-2-phenylindole dihydrochloride) was used to counterstain nuclei, and the labeling index was evaluated as the percentage of EdU-labeled nuclei in relation to the DAPI-stained nuclei. Therefore, 70–80 areas per experiment with each area containing 10–20 cells were selected and the number of EdU-labeled nuclei was counted by hand (31).

### Antibodies and reagents

The following antibodies were used: anti-AQP1 (ab9566, Abcam; 1:400), anti-AQP1 (AB2219, Merck Millipore; 1:400), anti-β-actin (A2228, Sigma-Aldrich; 1:10 000), anti-Caspase 3 (9662S, Cell Signaling Technology, Danvers, MA, USA; 1:500), anti-CD3 (ab16669, Abcam; 1:400), anti-cleaved Caspase 3 (9661S, Cell Signaling Technology; 1:500), anti-CathepsinD (69854, Cell Signaling Technology; 1:400), anti-Galectin 3 (14–5301-82, Invitrogen; 1:400), anti- Gc-globulin (also known as VDBP, A0021, Dako A/S, Glostrup, Denmark; 1:500), anti- Transferrin (A0061; Dako;1:500), anti-Ki-67 (14-5689-82, eBioscience, San Diego, CA, USA; 1:500), anti-Kim-1 (AF3689-SP, R&D Systems, Minneapolis, MN, USA; 1:400), anti-Lamp1 (ab24170, Abcam; 1:400), anti-Lipocalin-2 (AF1857, R&D Systems; 1:400), anti-LC3 (PM036, MBL International, Woburn, MA, USA; 1:100), anti-megalin (also known as LRP2, sc-16478, Santa Cruz Biotechnology, Dallas, TX, USA; 1:1000), anti-megalin (1:1000) that was kindly provided by P. Verroust and R. Kozyraki (INSERM, Paris, France), anti-Na^+^/K^+^-ATPase subunit α1 (C464.6, Merck Millipore; 1:200), anti-PCNA (M0879, Dako; 1:500), anti-p62 (ab56416, Abcam; 1:400), anti-p62 (ab109012, Abcam; 1:400), anti-Rab5 (18211, Abcam; 1:400), anti-Rab11 (3539, Cell Signaling Technology; 1:400) and mouse anti-α-tubulin (T5168, Sigma-Aldrich; 1:1000). Compounds included Bafilomycin (ALX-380-030, Enzo Life Sciences, Farmingdale, NY), Dynasore (D7693, Sigma-Aldrich) and 4′,6-diamino-2-phenylindole dihydrochloride (DAPI; D1306, Thermo Fischer Scientific; 1:1000).

### Data analysis and statistics

The plotted data were presented as mean ± standard error of the mean (SEM). Differences between experimental groups were evaluated using one-way analysis of variance (ANOVA) followed by Dunnet’s multiple comparison test or paired or unpaired two tailed Student’s *t*-test, when appropriate, and as indicated in the figure legends. Outliers were identified using the ROUT (*Q* = 1%) method. The sample size of each experimental group is described in the figure legends. The levels of statistical significance are indicated by symbols, and the *P*-values are indicated in the figure legends along with the statistical tests. All experiments reported here were performed at least three times independently, unless otherwise indicated in the figure legends. GraphPad Prism software v. 8.4.3 (GraphPad software) was used for performing all statistical analyses.

## Supplementary Material

HMG-2021-08853_R1_Krohn_SupplMat_ddac033
