# Supplementary material for: Multisystem involvement, defective lysosomes and impaired autophagy in a novel rat model of nephropathic cystinosis
Source: Hum Mol Genet. 2022 Feb 8;31(13):2262–78. doi: 10.1093/hmg/ddac033 (PMC9262394; doi:10.1093/hmg/ddac033)
Supplement: HMG-2021-08853_R1_Krohn_SupplMat_ddac033 [file hmg-2021-08853_r1_krohn_supplmat_ddac033.zip › HMG-2021-08853_R1_Krohn_SupplMat_ddac033.pdf]

HMG-2021-00853.R1

SUPPLEMENTARY MATERIAL

Multisystem Involvement, Defective Lysosomes, and Impaired Autophagy in a Novel Rat Model of Nephropathic Cystinosis

Patrick Krohn[1], Laura Rita Rega[2], Marianne Harvent[1], Beatrice Paola Festa[1], Anna Taranta[2], Alessandro Luciani[1], Joseph Dewulf[3,4], Alessio Cremonesi[5], Francesca Diomedi Camassei[6], James V. M. Hanson[7], Christina Gerth-Kahlert[7], Francesco Emma[2,8], Marine Berquez[1#] and Olivier Devuyst[1,9#*]

Supplementary Methods and References

Supplementary Figures

Supplementary Tables

SUPPLEMENTARY METHODS

Urine amino acids

LC-MS/MS analysis of urinary amino acids and related metabolites was performed and adapted using the Waters^{TM} Kairos^{TM} Amino Acid Kit (stable isotope dilution assay) in the Laboratory of Inherited Metabolic Diseases/Biochemical Genetics (Cliniques universitaires Saint-Luc, UCLouvain, B-1200, Bruxelles). Prior to LC-MS-MS analysis, proteins were precipitated using a sulfosalicylic acid solution containing stable-isotope labeled internal standards. Subsequently, the compounds containing primary and secondary amines were derivatized after reaction with 6-aminoquinolyl-N-hydroxysuccinimidyl carbamate (AccQTag^{TM} Reagent). The diluted derivatized samples were injected and eluted by reverse phase liquid chromatography using a C18 column (CORTECS C18 UPLC, 2.1 x 150 mm, particle size 1.6 µm, ref 186007096, Waters corporation, Wexford, Ireland) heated at 60°C on an Acquity UPLC-I-Class system (Waters Corporation, Wexford, Ireland). The mobile phases A[Water, 0.1 % formic acid] and B [Acetonitrile, 0.1 % formic acid] were used at a flow rate of 0.45 mL/min, using the following concentrations and linear gradients: 0-1min: 1% B; increase of B to 6% over 1 min; increase of B to 9.5% over 3 min; increase of B to 10.5% over 7.5 min; increase of B to 95% over 1 min; 95% B from 13.5 to 14.2 min; decrease of B to 1% over 0.1 min and 1% B from 14.3 to 16 min. Multiple Reaction Monitoring (MRM) analyses were performed using a Xevo^{TM} TQ-S micro mass spectrometer in positive electrospray ionization (ESI). The ESI spray voltage was 2000 V. The cone gas flow rate was 20L/h, desolvation gas flow 1000L/h and desolvation temperature 500°C. Stable-isotope labelled internal standards were used and calibration curves were made for each compound.

Liver function parameters

Blood samples were collected at sacrifice and liver enzymes including aspartate-aminotransferase (AST), alanine-aminotransferase (ALT), alkaline phosphatase (ALP) and gamma-glutamyl transferase (GGT) were measured by the Appia Laboratory (Rome, Italy).

Immunofluorescent image quantification

Numbers of "CD3^{+} cells per field" or "PCNA^{+} or Ki-67^{+} nuclei per tubule": The number of the CD3 or PCNA or Ki-67-positive structures per field were manually counted.

<u>Mean fluorescence intensity</u>: For all measurement of "mean fluorescence intensity", as previously described (1), an outline of each tubule or cell was drawn by hand using FIJI (Image J), and the mean

fluorescence intensity recorded. The number of tubules and cells taken for each analysis is stated in the figure legends.

Puncta per cell: The pipeline "Speckle counting" in CellProfiler™ was used to quantify the number of Cy5-β-lactoglobulin$^+$ puncta surrounding nuclei and to perform per-nuclei aggregate measurements (eg. the number of dots/nucleus), as previously described (1). In short, the images were converted to greyscale using the module "ColorToGray". Next, the "IdentifyPrimaryObjects" module was used to identify nuclei and dots. After, each cell was identified using the module "IdentifySecondaryObjects". "MaskObject" and "RelateObject" modules were used to establish a parent-child relationship between the cells and the dots, identified as masked objects.

Lysosomal area per tubule: Images acquired by confocal microscopy were first deconvolved using Huygens (SVI) software in order to increase object resolution, to improve the signal, and correct the noise. The final images were loaded into FIJI (Image J), followed by a colour deconvolution identifying the Lamp1 signal. Measurement of the tubule area and the analysis of particles within that area allowed the quantification of total lysosomal area per tubule.

Lysosomal vesicle diameter in rPTCs: Z-stack images acquired by confocal microscope were first deconvolved using Huygens (SVI) software in order to increase object resolution in x, y, and z, to improve the signal, and correct the noise. The final z-stack images were loaded into the Imaris software to complete the 3D reconstruction of Lamp1-positive vesicles. The open-source cell image analysis software CellProfiler™ , in particular the specific module "Measure-Object-Intensity-Distribution" was used to quantify the vesicle diameter as previously described (2).

Bone turnover marker analysis

Blood samples were taken from the rats at the indicated time points and in both sexes. Serum or plasma was used for the analysis of bone turnover markers. C-terminal telopeptide of type I collagen (CTX-I; Rat-Laps (CTX-I) EIA (Immunodiagnostic Systems, Boldon, UK)), tartrate-resistant acid phosphatase (TRAcP 5b ; RatTRAP™ ELISA (Immunodiagnostic Systems)), N-terminal propeptide of type I procollagen (P1NP; PINP EIA (Immunodiagnostic Systems, Boldon, UK)), parathyroid hormone (PTH; Rat Intact PTH EIA (Immunotopics Inc, San Clemente, CA, USA)), C-terminal Fibroblast growth factor 23 (FGF23; Mouse/Rat FGF-23 (C-Term) ELISA Kit (Immunotopics Inc)) and 1,25-dihydroxyvitamin D3 (1,25 DHVD3; Rat 1, 25 dihydroxyvitamin D3 (1,25 DHVD3)

ELISA Kit (MyBioSource, San Diego, CA, USA)) were measured by ELISA or EIA kits, according to the manufacturers' instructions.

Micro-computed tomography and Bone Microstructural Analyses

Femurs were excised and cleaned to remove soft tissues. Dissected femurs were scanned using a Quantum GX microCT Imaging System (Perkin Elmer, Hopkinton, MA, USA), with a focal size of 20 mm, a tube voltage of 90 kV, a tube current of 100 μA and an isotropic voxel size of 40 μm. For calibration of bone data to bone mineral density (BMD) values, a commercially available calcium hydroxyapatite phantom (QRM GmbH, Moehrendorf, Germany) was scanned using the same settings. Reconstruction and processing of the images for bone analysis was performed using commercially available software (Analyze 12.0, AnalyzeDirect, Inc., Overland Park, KS)(3). Proximal to the distal femoral growth a 200-slice thick volume of interest (VOI) was identified, beginning where the epiphyseal cap structure completely disappeared. For BMD analysis the average grey level intensity was measured for the different calcium hydroxyapatite inserts of the phantom and a linear calibration was derived between grey level intensity and BMD.

Optical coherence tomography and slit lamp microscopy

At each time point, selected animals were anesthetized with intraperitoneal injections of Ketamine (100 mg/ml) and Xylazine (20 mg ml^{-1}). Immediately after, both anterior eyes of each rat were examined using Optical Coherence Tomography (OCT; SPECTRALIS HRA + OCT2 with Anterior Segment Objective, Heidelberg Engineering, Heidelberg, Germany). For each eye, a 15º x 10º volume scan comprising 81 b-scans separated by 69 μm was acquired in High Resolution mode with nine Automatic Real-time Tracking scans averaged; in all rats, this was sufficient to obtain OCT coverage of the entire corneal surface. OCT was followed by slit lamp biomicroscopy and photography (Slit lamp BQ 900, Haag-Streit Diagnostic, Köniz, Switzerland). These examinations enabled us to assess and document the presence and location of cystine crystals. Animals were then immediately sacrificed under anesthesia by bleeding.

SUPPLEMENTARY REFERENCES

1. Berquez, M., Gadsby, J.R., Festa, B.P., Butler, R., Jackson, S.P., Berno, V., Luciani, A., Devuyst, O. and Gallop, J.L. (2020) The phosphoinositide 3-kinase inhibitor alpelisib restores actin organization and improves proximal tubule dysfunction in vitro and in a mouse model of Lowe syndrome and Dent disease. *Kidney Int*, **98**, 883–896.

2. Festa, B.P., Chen, Z., Berquez, M., Debaix, H., Tokonami, N., Prange, J.A., Hoek, G. van de, Alessio, C., Raimondi, A., Nevo, N., *et al.* (2018) Impaired autophagy bridges lysosomal storage disease and epithelial dysfunction in the kidney. *Nat Commun*, **9**, 161.

3. Daryadel, A., Natale, L., Seebeck, P., Bettoni, C., Schnitzbauer, U., Gassmann, M. and Wagner, C.A. (2019) Elevated FGF23 and disordered renal mineral handling with reduced bone mineralization in chronically erythropoietin over-expressing transgenic mice. *Sci Rep*, **9**, 14989.

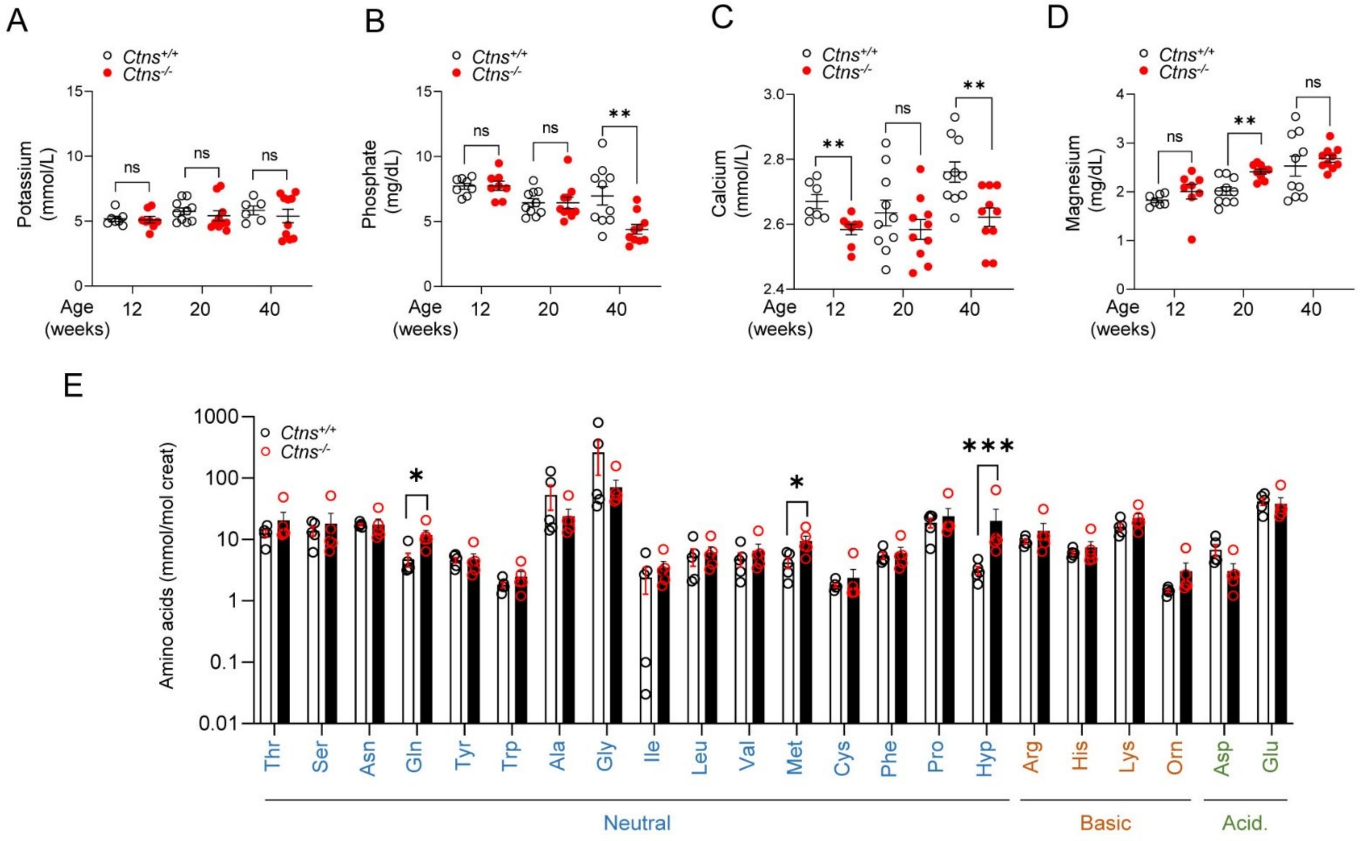

Supplementary Figure 1: Plasma electrolytes and urinary amino acid levels in *Ctns* rats. Measurement of (**A**) potassium, (**B**) phosphate, (**C**) calcium and (**D**) magnesium in plasma samples from *Ctns* rats at 12, 20 and 40 weeks of age (n=8 rats per group at 12 weeks and n=10 rats per group at 20 and 40 weeks for phophate, calcium and Magnesium; n= 7, 10 and 6 *Ctns*$^{+/+}$ and n=8, 10 and 10 *Ctns*$^{-/-}$ rats at 12, 20 and 40 weeks respectively for potasium, excluding hemolysed samples). (**E**) Relative urinary concentration of amino acids in urine derived from 20 week old *Ctns* rats (n= 5 rats per group). Ala, alanine; Arg, arginine; Asn, asparagine; Asp, aspartate; Cys, cysteine; Gln, glutamine; Glu, glutamate; Gly, glycine; His, histidine; Hyp, hydroxyproline; Ile, isoleucine; Leu, leucine; Lys, lysine; Met, methionine; Orn, ornithine; Phe, phenylalanine; Pro, proline; Ser, serine; Thr, threonine; Trp, tryptophan; Tyr, tyrosine; Val, valine. Each dot of the graph represents one rat. Plotted data represent mean ± SEM. Each dot represents one rat. Two-tailed unpaired Student's t-test, *P < 0.05, **P < 0.01 and ****P* < 0.001 relative to *Ctns*$^{+/+}$ rats.

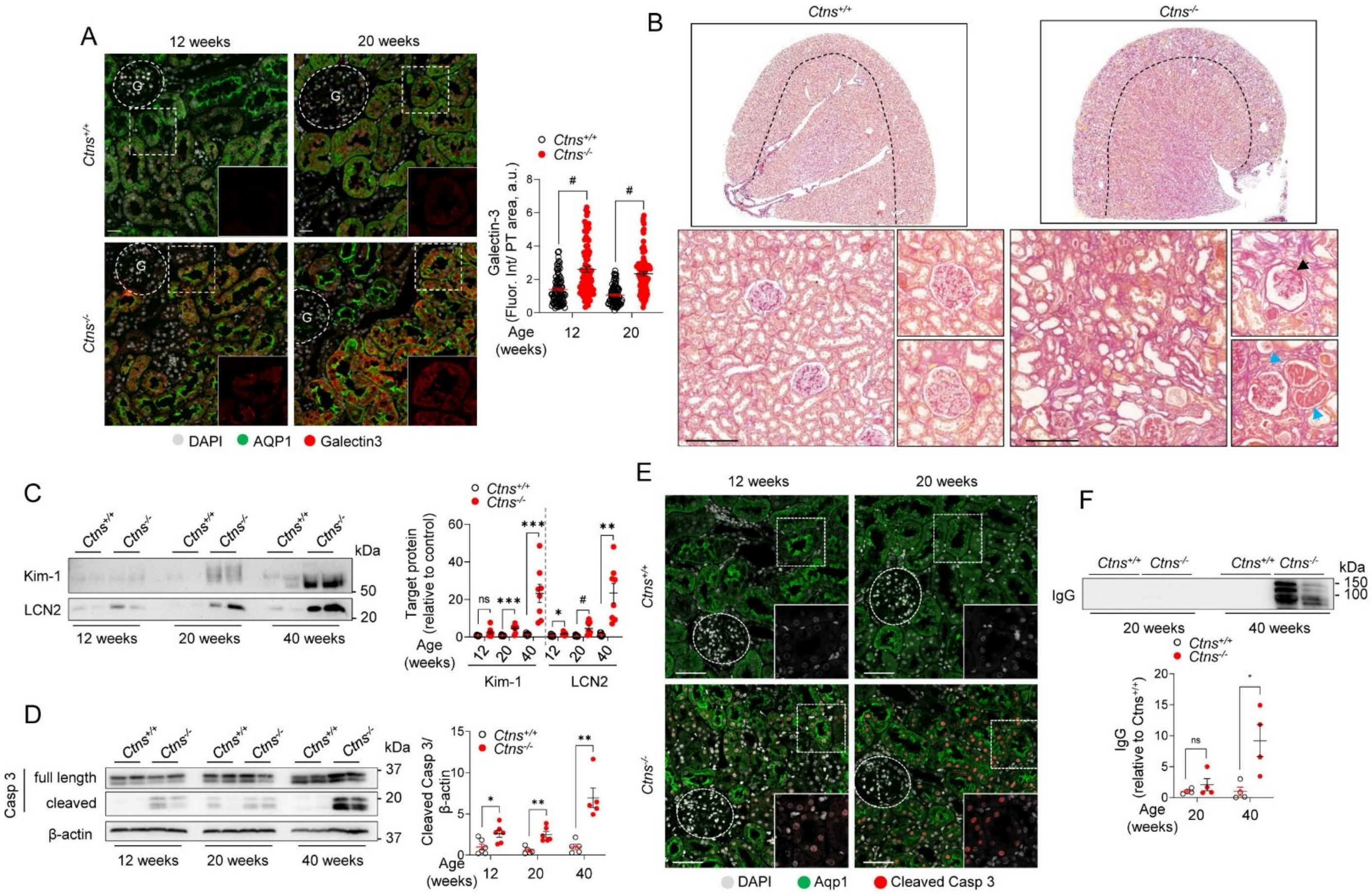

Supplementary Figure 2: Kidney lesions in cystinotic rats. (**A**) Representative confocal micrographs and quantification of the mean fluorescence intensity of Galectin-3 (red) at different ages (n= 87 tubules per group, pooled from three rats). Each dot of the graph represents one tubule. (**B**) Representative images of Picro-sirius Red staining in kidneys from 72 week-old rats. The black dotted line delaminates the cortex from the medulla. In enlarged boxes, high magnifications of the corresponding section are shown. Insets: high magnification of the corresponding section displaying glomerular lesions (top panel, black arrow head) and protein casts (bottom panel, blue arrow head) in *Ctns*$^{-/-}$ animals. (**C**) Western blotting and densitometry analyses of Kim-1 and LCN2 in the urine from *Ctns* rats at different ages (n= 8 rats per group). (**D**) Western blotting and densitometry analysis of full length and cleaved Casp 3 protein levels in whole-kidney lysates from *Ctns* rats at different ages (n= 6, 5, 5 *Ctns*$^{+/+}$ and n= 6, 6, 5 *Ctns*$^{-/-}$ rats at 12, 20 and 40 weeks of age, respectively). β-actin was used as loading control. (**E**) Representative confocal micrographs of cleaved Caspase 3 (Casp 3) at different ages. (**F)** Representative western blotting and densitometry quantification of IgG in urine derived from 20 and 40 week old *Ctns* rats (n=4 rats per group). Loads were normalized to 1µg of creatinine. Proximal tubules labelled by AQP1 (green) and nuclei counterstained with DAPI (grey) in (**A** and **E**). Scale bars: 20 µm in (**A**), 500 µm in (**B)** and 50 µm in (**E**). Plotted data represent mean ±SEM. Two-tailed unpaired Student's t-test, *P < 0.05, **P < 0.01 and #P < 0.0001 relative to *Ctns*$^{+/+}$ rats, G, Glomerulus.

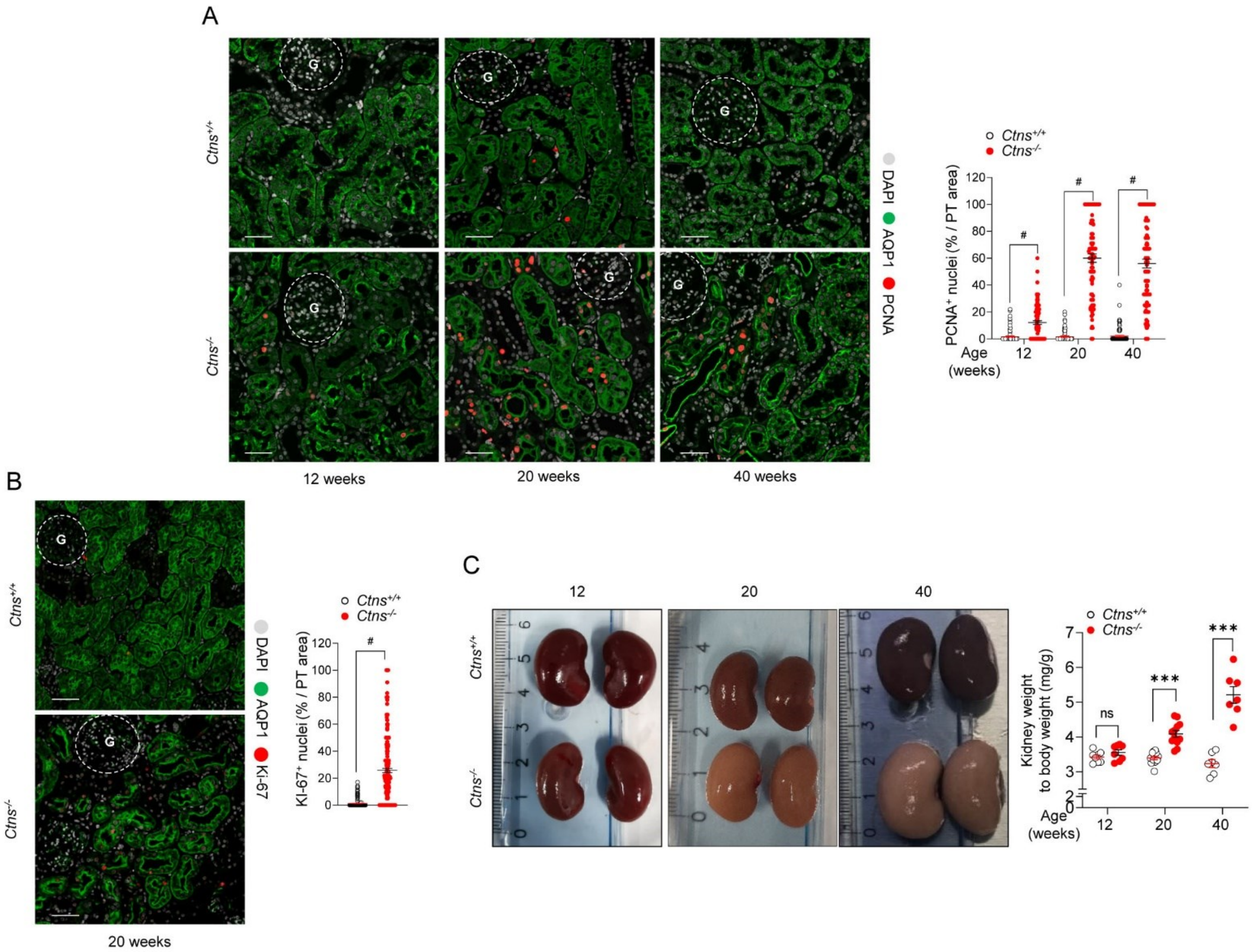

Supplementary Figure 3: Increased proliferation over time in *Ctns*$^{-/-}$ proximal tubules. (**A**) Representative confocal micrographs and quantification of the percentage of PCNA^{+} (red) nuclei in AQP1^{+} (green) proximal tubules of *Ctns* rat kidneys at different ages (n=90 tubules per condition, pooled from 3 rats per group). (**B**) Representative confocal micrographs and quantification of the percentage of Ki-67^{+} (red) nuclei in AQP1^{+} (green) proximal tubules of *Ctns* rat kidneys (n= 300 *Ctns*$^{+/+}$ and n= 250 *Ctns*$^{-/-}$ tubules, pooled from 5 rats per group). Nuclei counterstained with DAPI (grey) in (**A)** and (**B**). Each dot of the graphs represent one tubule in (**A**) and (**B).** Scale bars: 50 µm in (**A)** and (**B).** (**C**) Representative pictures of *Ctns* rat kidneys at 12, 20 and 40 weeks and ratio of kidney weight to body weight (n=8, 12 and 7 rats per group at 12, 20 and 40 weeks of age, respectively). Each dot of the graph represents the average of both kidneys derived from one rat. Plotted data represent mean ±SEM. Two-tailed unpaired Student's t-test, ***$P <$ 0.001, and #$P < 0.0001$ relative to *Ctns*$^{+/+}$ rats. G, Glomerulus.

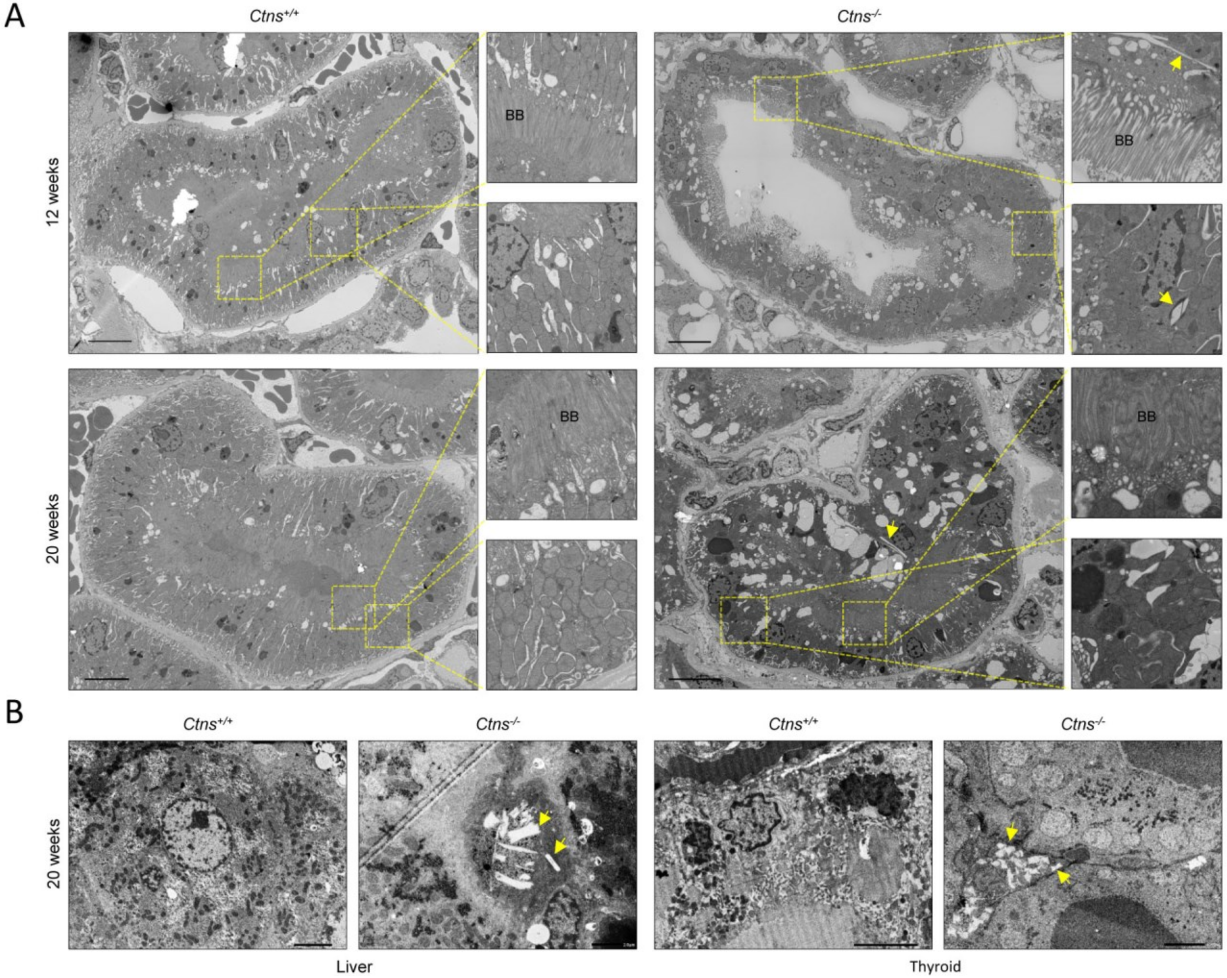

Supplementary Figure 4: Crystal formation in multiple tissues of *Ctns*$^{-/-}$ rats. (**A**) Representative electron micrographs of proximal tubules derived from 12 and 20 week old *Ctns* rat kidneys. Arrowheads indicate the presence of needle shaped crystals. Insets: high magnification of the corresponding area. (**B**) Representative electron micrographs of liver and thyroid cells of *Ctns* rats at 20 weeks of age. Arrowheads indicate the presence of needle shaped crystals. Scale bars: 10 µm in (**A)** and 2 µm in **(B).** BB, Brush border.

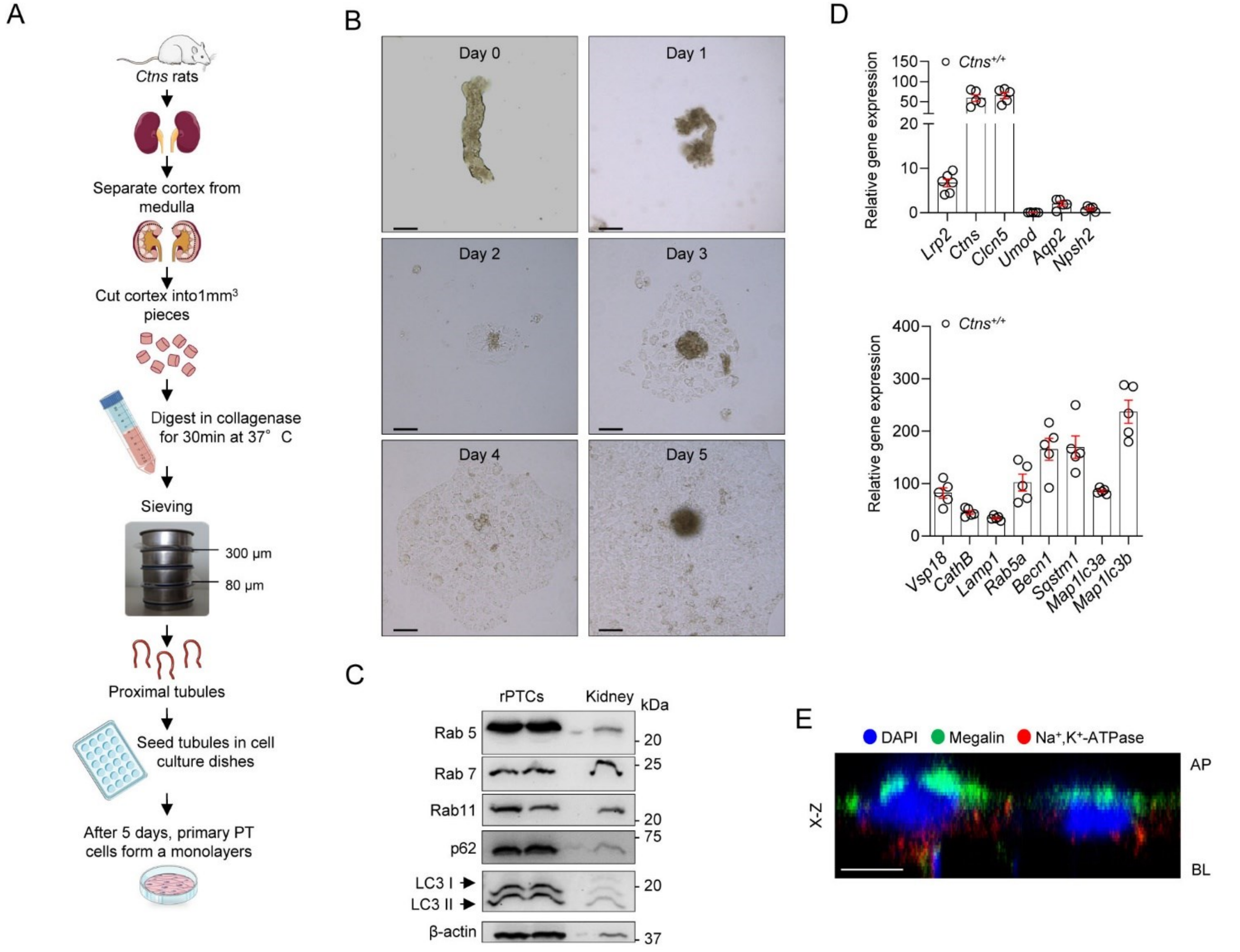

Supplementary Figure 5: Primary proximal tubule cells derived from rat kidneys express critical aspects of proximal tubule function. (**A**) Detailed workflow of the strategy used to develop primary proximal tubule cells derived from *Ctns* rat kidneys (rPTCs). (**B**) Representative pictures of time dependent PT cell cultivation. (**C**) Representative western blots of the endolysosomal and autophagy markers in rPTCs derived from *Ctns*$^{+/+}$ kidneys. (**D**) The mRNA level of PT, endolysosomal and autophagy markers were analyzed by RT-qPCR in *Ctns*$^{+/+}$ rPTCs. Gene target expression normalized to *Gapdh* and relative to *Ctns*$^{+/+}$ tubules (n = 5 biologically independent experiments). (**E**) Representative confocal micrographs of X–Z side view of a z-stack performed on *Ctns*$^{+/+}$ rPTCs immunostained with Megalin (green) and Na^+,K^+-ATPase (red). Nuclei counterstained with DAPI (blue). Plotted data represent mean ±SEM. Scale bars: 100 µm in (**A**) and 5 µm in (**E**).

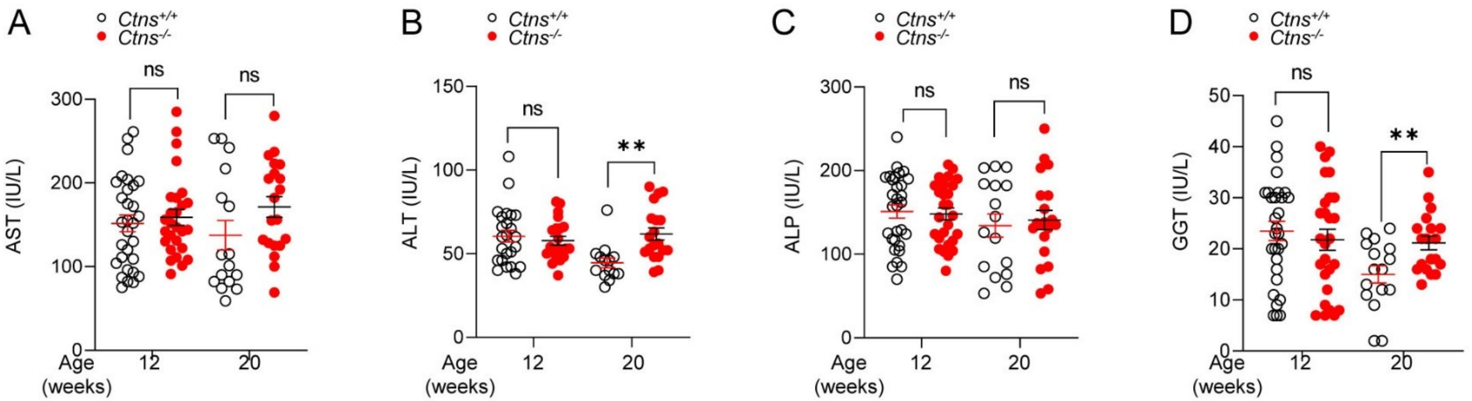

Supplementary Figure 6: Hepatotoxicity markers in *Ctns* rats. Levels of (**A**) aspartate-aminotransferase (AST), (**B**) alanine-aminotransferase (ALT), (**C**) alkaline phosphatase (ALP), and (**D**) gamma-glutamyl transferase (GGT) measured from *Ctns* rat plasma at 12 and 20 weeks of age (n= 30 $Ctns^{+/+}$ and n= 27 $Ctns^{-/-}$ rats at 12 weeks; n= 16 $Ctns^{+/+}$ and n= 20 $Ctns^{-/-}$ rats at 20 weeks). Plotted data are mean ± SEM. Each dot represents one rat. Two-tailed unpaired Student's t-test, $^{**}P < 0.01$, relative to $Ctns^{+/+}$ rats.

Supplementary Table 1: Blood electrolytes in *Ctns* rats.

	12 weeks male		12 weeks female		20 weeks male		20 weeks female		40 weeks male		40 weeks female	
	Ctns$^{+/+}$ n=4	*Ctns*$^{-/-}$ n=4	*Ctns*$^{+/+}$ n=5	*Ctns*$^{-/-}$ n=5	*Ctns*$^{+/+}$ n=5	*Ctns*$^{-/-}$ n=5	*Ctns*$^{+/+}$ n=5	*Ctns*$^{-/-}$ n=5	*Ctns*$^{+/+}$ n=5	*Ctns*$^{-/-}$ n=5	*Ctns*$^{+/+}$ n=5	*Ctns*$^{-/-}$ n=5
Calcium (mmol/L)	2.63 ± 0.01	2.57 ± 0.03	2.7 ± 0.03	2.6 ± 0.01*	2.56 ± 0.03	2.58 ± 0.05	2.71 ± 0.05	2.58 ± 0.04*	2.77 ± 0.06	2.61 ± 0.04	2.75 ± 0.04	2.63 ± 0.05
Magnesium (mg/dL)	1.46 ± 0.27	1.95 ± 0.32	1.91 ± 0.04	2.06 ± 0.08	2.13 ± 0.11	2.63 ± 0.17*	1.91 ± 0.11	2.4 ± 0.07**	3.09 ± 0.15	2.79 ± 0.11	1.96 ± 0.06	2.6 ± 0.11***
Phosphate (mg/dL)	8.27 ± 0.09	8.08 ± 0.47	7.16 ± 0.2	7.41 ± 0.54	6.93 ± 0.41	7.3 ± 0.66	5.96 ± 0.37	5.59 ± 0.17	8.9 ± 0.56	5.3 ± 0.50***	5.05 ± 0.33	3.52 ± 0.11**
Potassium (mmol/L)	5.42 ± 0.46	5.31 ± 0.51	5.01 ± 0.08	4.92 ± 0.1	5.7 ± 0.35	5.29 ± 0.58	5.82 ± 0.41	5.54 ± 0.57	5.38 ± 0.44	5.03 ± 0.8	6.28 ± 0.42	5.76 ± 0.72

All the measurements were performed on *Ctns*$^{+/+}$ and *Ctns*$^{-/-}$ male and female rats matched per age. Two-tailed unpaired Student's t-test was applied between genotypes at the indicated time points. $P < 0.05$, **$P < 0.01$, ***$P < 0.001$ relative to *Ctns*$^{+/+}$ rats.

Supplementary Table 2: Hepatotoxicity markers in *Ctns* rats.

	12 weeks male		12 weeks female		20 weeks male		20 weeks female	
	Ctns$^{+/+}$ n=15	*Ctns*$^{-/-}$ n=12	*Ctns*$^{+/+}$ n=15	*Ctns*$^{-/-}$ n=15	*Ctns*$^{+/+}$ n=10	*Ctns*$^{-/-}$ n=10	*Ctns*$^{+/+}$ n=6	*Ctns*$^{-/-}$ n=10
AST (IU/L)	166 ± 10.6	156 ± 11.5	137 ± 16.2	163 ± 16.2	160 ± 35.8	162 ± 18.7	124 ± 18.7	181 ± 16.7*
ALT (IU/L)	60 ± 2.4	65 ± 3.5	56 ± 5.8	55 ± 3.2	55 ± 7.8	81 ± 7.4*	42 ± 2.5	50 ± 2.3
ALP (IU/L)	168 ± 8.6	162 ± 7.9	135 ± 12.4	132 ± 11.2	140 ± 22.0	131 ± 17.7	131 ± 18.5	151 ± 15.0
GGT (IU/L)	25 ± 2.7	20 ± 2.6	22 ± 2.7	24 ± 3.3	13 ± 2.8	20 ± 2.2	16 ± 2.1	22 ± 1.6

AST, Aspartate-aminotransferase; ALT, Alanine-aminotransferase; ALP, Alkaline phosphatase; GGT, Gamma-glutamyl transferase. Measurements were performed on *Ctns*$^{+/+}$ and *Ctns*$^{-/-}$ rats matched for age and sex. Two-tailed unpaired Student's t-test was applied between genotypes at the indicated time points. *$P < 0.05$, relative to *Ctns*$^{+/+}$ rats.

Supplementary Table 3: Bone turnover markers in *Ctns* rats.

	24 weeks male		24 weeks female		36 weeks male		36 weeks female	
	Ctns$^{+/+}$ n=5	*Ctns*$^{-/-}$ n=5	*Ctns*$^{+/+}$ n=5	*Ctns*$^{-/-}$ n=5	*Ctns*$^{+/+}$ n=5	*Ctns*$^{-/-}$ n=5	*Ctns*$^{+/+}$ n=5	*Ctns*$^{-/-}$ n=5
PTH (ng/ml)	983 ± 167	620 ± 129	490 ± 67.6	437 ± 88.9	974 ± 256	547 ± 167	307 ± 49	392 ± 97.3
FGF23 (ng/ml)	365 ± 26.6	420 ± 33	372 ± 34.9	356 ± 38.8	311 ± 17.6	415 ± 34*	357 ± 25.9	264 ± 29.5
TRAcP 5b (ng/ml)	2.62 ± 0.21	5.2 ± 0.23$^{\#}$	4.94 ± 0.68	8.4 ± 0.74**	-	-	-	-
P1NP (ng/ml)	0.82 ± 0.07	0.99 ± 0.12	0.58 ± 0.06	0.47 ± 0.05	-	-	-	-
CTX (ng/ml)	36.5 ± 2.7	45.8 ± 4.23	22.8 ± 1.17	33.6 ± 2.0**	-	-	-	-
1,25(OH)2 Vit D3 (ng/ml)	197 ± 9.72	175 ± 7.63*	212 ± 6.63	187 ± 3.94	-	-	-	-

PTH, Parathyroid hormone; FGF23, Fibroblast growth factor 23; 1,25(OH)2 Vit D3, 1-a hydroxylation of vitamin D3; TRAcP 5b, Tartrate-resistant acid phosphatase; CTX I, C-terminal telopeptide of type I collagen; P1NP, N-terminal propeptide of type I procollagen. All the measurements were performed on *Ctns*$^{+/+}$ and *Ctns*$^{-/-}$ male and female rats matched per age. Two-tailed unpaired Student's t-test was applied between genotypes at the indicated time points. *$P < 0.05$ and **$P < 0.01$ relative to *Ctns*$^{+/+}$ rats.

Supplementary Table 4: Microarchitectural parameters of the femurs determined by microCT in *Ctns* rats at 56 weeks.

	BMD (mg Ha/cm^3)	Tb. BMD (mg Ha/cm^3)	BV/TV (in %)	Ct.Ar (mm^2)	Tb.N (1/mm)	Tb.Th (mm)	Tb.Sp (mm)	Conn.D (1/mm^3)	SMI
Ctns$^{+/+}$ (n=3)	1031 ± 17	673 ± 7	53.4 ± 6.2	6.6 ± 0.3	2.39 ± 0.1	0.2 ± 0.02	0.28 ± 0.02	35.4 ± 6.3	0.18 ± 0.04
Ctns$^{-/-}$ (n=3)	1088 ± 18	624 ± 17	29.5 ± 3.8*	4.3 ± 0.2**	2.01 ± 0.1*	0.19 ± 0.02	0.45 ± 0.03**	39.9 ± 5	0.24 ± 0.02

Bone mineral density (BMD, mg Hydroxyapatite/cm3); Trabecular BMD (Tb. BMD, mg Hydroxyapatite/cm3); Bone volume fraction (BV/TV, %); Cortical bone area (Ct.Ar, mm2); Trabecular number (Tb.N, 1/mm); Trabecular thickness (Tb.Th, mm); Trabecular separation (Tb.Sp, mm), Connectivity density (Conn.D, 1/mm3); Structure model index (SMI). Plotted data represent mean ± SEM. Two-tailed unpaired Student's t-test, *P < 0.05 and ***P* < 0.01 relative to *Ctns*$^{+/+}$ rats.

Supplementary Table 5: Primer pairs for gene expression analysis in rat kidneys.

Gene name	Forward primer (5'-3')	Reverse primer (5'-3')
Ctns	TGAACTTGACAGGCTTCGTG	GCGACTGCATGTAAGCTGAA
Gapdh	ACCACAGTCCATGCCATCA	TCAGCTCTGGGATGACCTTG
Cd3g	ATGTATCAGTGCCGAGGAGC	AGCTCTTGACTGGCGAACTC
Ccl19	GGTGCTAACGATGCGGAAGA	CGGATGATGCGTTCTACCCA
Lgals3	TCGCCTTCCACTTTAACCCC	GGCTTCAACCAGGACCTGTA
Tlr4	TGTATCGGTGGTCAGTGTGC	CAGCTCGTTTCTCACCCAGT
Col6a1	GCCACAACTTCGAAACCACC	GCTGGAACTGAAGAGACGCT
Col1a1	GTACATCAGCCCAAACCCCA	GTGATGTTCTGGGAGGCCTC
Fn1	ATCCCACCAACAACCCAGTG	GCCCCGGAACATGAGGATAG
Col3a1	TGGAGGTGAAAAGTCTGGCG	AGGTCTCTGAATTCCGAGC
Vim	TGCCCTTGAAGCTGCTAACT	AATCCTGCTCTCCTCCCCTT
Ccna2	GTTTTGAATCACCCCATGCT	AGTGATGTCTGGCTGCCTCT
Ccnb2	TGCAGTCCATCAATCCACAT	GAAGGAACCGGTCCATGATA
Cdk1	CTGGCCAGTTCATGGATTCT	CCGAAATCTGCCAGTTTGAT
Clcn5	GGGCAAGTATCCTGTTGTCG	CAGAGCTTGGAGGAGTCCAG
Lrp2	CATGGACATCGGTGTGTCTC	GGCCACTTTGGAAGTGTTGT
Cubn	CCCTCACAAGACTGCTCACA	TGCACTTGCGAGTTTCTGTT
Slc5a2	TCTTTGTGCCCCGTGTTAAT	AGTGTACCCGGCAGAAGATG
Scl34a1	TCAGTATTGAGCGGGCCTAC	GCCCGAGATGTTGAAGAAGA
Aqp2	AGAGCTCTTCCTGACCATGC	GGAGCAACCGGTGAAATAGA
Umod	CAGGAAACTCCGACCTTGTC	GTGTGATGGGACCCAAGTTC
Npsh2	TTTCTACCCTGCCTGGACAC	GAAGAAGGGAGGCATTTTCC
Cathb	CCAGTGGAGGGTGCTTTTAC	TTTGCTACCAGCCAGTAGGG
Vps18	TCTGGCCTACCTGGAACAAG	CAGGTCCACAGCCTCTTCAT
Lamp1	CACGACTGTGACCAGAGCAT	CTCCTTGCAGGAAAAACAGG
Rab5a	GGATACAGCTGGCCAAGAAC	AGGACTTGCTTGCCTTTGAA
Becn1	GTGCTCCTGTGGAATGGAAT	TGTCTGTCAGGGACTCCAGA
Sqstm1	ACCCCAACGTGATTTGTGAT	AAGGGGTTGGGAAAGATGAG
Map1lc3a	AGCGCTACAAGGGTGAGAAG	GGTTCACCAGCAGGAAGAAG
Map1lc3b	GCGATACAAGGGTGAGAAGC	CCATTCACCAGGAGGAAGAA
Lcn2	ACC GTC ACT TCC ATC CTC GT	CCA TGG CAA ACT GGT CGT AG
Havcr1	CAC TCA CTG CAG CGA TTC TC	ATG CTC CCA TGA TTT TGC CC
Cst3	TGG TCC CTT ACT TGT TCC CC	GGA GAA GAG AAC CAG GGG AC
Clu	GAA CCA GAG CTC ACC CTT CT	AAG AAA TGA GGC CGC TTG TG
Ccn1	CCT GGG TTT CTA GTG TGG GT	ACT GCG TTA CTG TCC ATC CA
